# Type I interferon signaling promotes early innate control of *Borrelia burgdorferi* infection

**DOI:** 10.1371/journal.ppat.1013996

**Published:** 2026-10-26

**Authors:** Raj Priya, Sajith Raghunandanan, Darko Mihaljica, Nada S. Alakhras, Mark H. Kaplan, X. Frank Yang

**Affiliations:** 1 Department of Microbiology and Immunology, Indiana University School of Medicine, Indianapolis, Indiana, United States of America; 2 Department of Public Health Genomics, Manipal School of Life Sciences, Manipal Academy of Higher Education, Manipal, India; Centre National de la Recherche Scientifique, FRANCE

## Abstract

*Borrelia burgdorferi*, the causative agent of Lyme disease, elicits a robust type I interferon (IFN-I) response that has been strongly associated with chronic inflammatory manifestations such as Lyme arthritis. Although IFN-I is known to be induced early during infection, its functional role in the initial stages of *B. burgdorferi* infection remains unclear. Here, we identify a protective role for IFN-I signaling in the early control of *B. burgdorferi*. At 5 days post-challenge, mice lacking the type I interferon receptor (IFNAR1) exhibited markedly elevated spirochetal burdens at the site of inoculation, indicating that IFN-I signaling contributes to restricting early bacterial expansion. By 10 days post-challenge, IFNAR1 deficiency resulted in significantly increased bacterial loads in the skin and a trend toward higher loads in heart tissues, while joint burdens remained unaffected. Mechanistically, IFN-I signaling enhanced macrophage uptake of *B. burgdorferi*, phagosomal processing, and intracellular killing, while also modulating inflammatory cytokine responses and promoting macrophage recruitment to the inoculation site. Conversely, pharmacological activation of IFN-I signaling augmented macrophage antimicrobial function and improved bacterial clearance. Together, these findings establish IFN-I as a key component of early host defense against *B. burgdorferi* and reveal a time-dependent role for IFN-I signaling in Lyme disease pathogenesis, with protective functions during early infection that contrast with its pathogenic effects at later stages.

## Author summary

Lyme disease is caused by the spirochete *Borrelia burgdorferi*, which is transmitted to humans through the bite of infected ticks. The spirochete must evade the host’s immune defenses to establish infection in the skin, persist, and disseminate to other tissues to cause disease. Type I interferons (IFN-I) are best known for their antiviral roles, but they are also strongly induced during Lyme disease. While IFN-I responses have been linked to the development of Lyme arthritis in later stages of infection, their role during the earliest phase of Lyme disease has remained unclear. In this study, we show that IFN-I signaling plays a protective role early after *B. burgdorferi* inoculation. Mice lacking the type I interferon receptor (IFNAR1) were unable to efficiently control bacterial growth at the site of inoculation. Mechanistically, IFN-I signaling enhanced the ability of macrophages to engulf, process, and kill *B. burgdorferi*, promoted inflammatory cytokine production, and supported the recruitment of macrophages to the site of inoculation. Importantly, short-term activation of IFN-I signaling enhanced bacterial clearance, whereas loss of this pathway impaired early immune control. Together, these findings reveal that IFN-I has time-dependent roles during Lyme disease, protecting the host during early-stage infection while contributing to inflammation at later stages. By defining the time-dependent role of IFN-I signaling in Lyme disease, our study advances understanding of how innate immune responses shape infection outcome and highlights potential strategies for enhancing early host defense.

## Introduction

Lyme disease, caused by the spirochete *Borrelia burgdorferi (B. burgdorferi)*, is the most prevalent vector-borne illness in the United States and Europe [1]. Each year, hundreds of thousands of cases are reported, underscoring its substantial public health burden [2,3]. Following transmission, *B. burgdorferi* establishes infection in the skin, where it evades early immune clearance and disseminates to distant tissues, including the joints, heart, and nervous system [4,5]. Disseminated infection can lead to severe clinical outcomes such as Lyme arthritis, Lyme carditis, and neuroborreliosis. The absence of a human vaccine, together with challenges in diagnosing and managing persistent disease, underscores the need to better understand the host–pathogen interactions that shape infection outcome. One of the key gaps lies in our understanding of the early innate immune response in the skin, where the initial encounter between host defenses and the spirochete influences whether *B. burgdorferi* is cleared, persists locally, or disseminates to distal tissues.

Type-I Interferons (IFN-I), classically defined by their roles in antiviral defense [6–8] are rapidly induced upon nucleic acid sensing and activate hundreds of interferon-stimulated genes (ISGs) that establish broad antimicrobial states [9,10]. IFN-I is now recognized to modulate immune responses during bacterial infections as well [11]. However, IFN-I responses exert complex and sometimes opposing effects during bacterial infections, with outcomes that vary depending on the nature of a pathogen, infection site, timing, and strength of induction. In some contexts, IFN-I supports antibacterial immunity by enhancing immune cell activation and increasing inflammatory cytokines that contribute to bacterial control [12–15]. However, IFN-I can also be detrimental to the host by suppressing protective immune responses through the induction of anti-inflammatory cytokines and inhibition of proinflammatory mediators and chemokines, or promoting apoptosis of macrophages and lymphocytes, resulting in increased bacterial dissemination [16–18].

*B. burgdorferi*, an extracellular spirochetal pathogen, elicits a robust IFN-I response in both mice and humans [19,20]. Much of this IFN-I induction occurs through pathways independent of MyD88- and TRIF-mediated Toll-like receptors (TLRs) [20–23], but is driven largely by intracellular sensing of spirochetal components through the cGAS–STING axis [24,25]. In addition, endosomal TLR7/8 sensing of *B. burgdorferi* RNA may also contribute to IFN-I production in certain host cells [19,26,27]. *B. burgdorferi*-induced IFN-I plays a pathogenic role in Lyme disease. Research to date has focused primarily on its effects during the later stages of infection, where elevated IFN-I levels correlate with increased inflammation and tissue damage, which contributes significantly to the development and severity of Lyme arthritis in mouse models [23,28–30]. In addition, IFN-I responses drive the abnormal accumulation of naïve B cells in lymph nodes, which may compromise the efficacy of the adaptive immune response [22]. In humans, elevated IFN-I responses have also been associated with disease severity, including persistent symptoms following Lyme neuroborreliosis [31] and cognitive deficits following antibiotic treatment [32].

Accumulated evidence indicates that during the early acute stage of infection, *B. burgdorferi* also induces a strong IFN-I response in mouse models, tick bite sites, and human erythema migrans lesions [33–36]. Transcriptional profiling of human erythema migrans (EM) lesions reveals that ISGs constitute a dominant portion of the inflammatory signature, preceding the onset of adaptive immunity [35]. Increased IFN‑I in serum and EM blister fluids correlates with disseminated disease in patients, and *B. burgdorferi* phagocytosis induces IFN-I production from macrophages, plasmacytoid dendritic cells (pDCs), and myeloid dendritic cells (mDCs) [26,34,35]. In murine infection models, *B. burgdorferi* triggers the expression of IFN-β and multiple ISGs in skin and blood within the first days of infection, and the magnitude of this early IFN-I response correlates with strain dissemination capacity [34]. Despite these observations, the role of IFN-I responses in host defense during the early stage of *B. burgdorferi* infection remains unclear.

To address this unresolved question, in this study, we investigated the role of IFN-I signaling in the early control of *B. burgdorferi* infection. Specifically, we evaluated its influence on phagocytosis, phagosomal processing, intracellular killing, production of proinflammatory cytokines, immune cell recruitment, and spirochetal burden at the site of inoculation. We demonstrate that IFN-I is important for effective early control of *B. burgdorferi* by enhancing bacterial uptake and augmenting macrophage-derived antimicrobial responses, thereby significantly reducing the bacterial burden during the initial phase of infection. These results reveal a previously underappreciated protective mechanism driven by IFN-I that coordinates effective early innate immunity. By identifying IFN-I as a central regulator of early phagocytic and antimicrobial responses, this work provides insights suggesting that therapeutic strategies designed to enhance IFN-I signaling during the initial stages of infection could strengthen host defense, limit bacterial burden, and potentially reduce the risk of post-treatment complications.

## Results

### Type I IFN signaling controls early *B*. *burgdorferi* burden

To determine the role of IFN-I signaling during early *B. burgdorferi* infection, we quantified spirochetal burden in wild-type (WT) and *Ifnar1^−/^^−^* mice at 5- and 10-days post- inoculation (DPI) in the skin, heart, and joints using quantitative PCR (qPCR) (Fig 1). At 5 DPI, *Ifnar1^−/^^−^* mice showed a significantly higher *B. burgdorferi* burden in the skin (2.1-fold, at the site of inoculation), compared with WT controls (Fig 1A). At 10 DPI, the difference in bacterial burden in the skin became more pronounced, with *Ifnar1^−/^^−^* mice significantly exhibiting a 5.3-fold higher spirochetal burden than WT mice (Fig 1B, left). A 3.7-fold higher spirochetal burden in the heart, although the difference did not reach statistical significance, was also observed (Fig 1B, center). In contrast, spirochetal burdens in joint tissues were comparable between WT and *Ifnar1^−/^^−^* mice (Fig 1B, right). These findings suggest that IFN-I signaling contributes to early control of *B. burgdorferi* at the inoculation site, which may in turn limit subsequent dissemination to distal tissues such as skin and heart.

**Fig 1:**
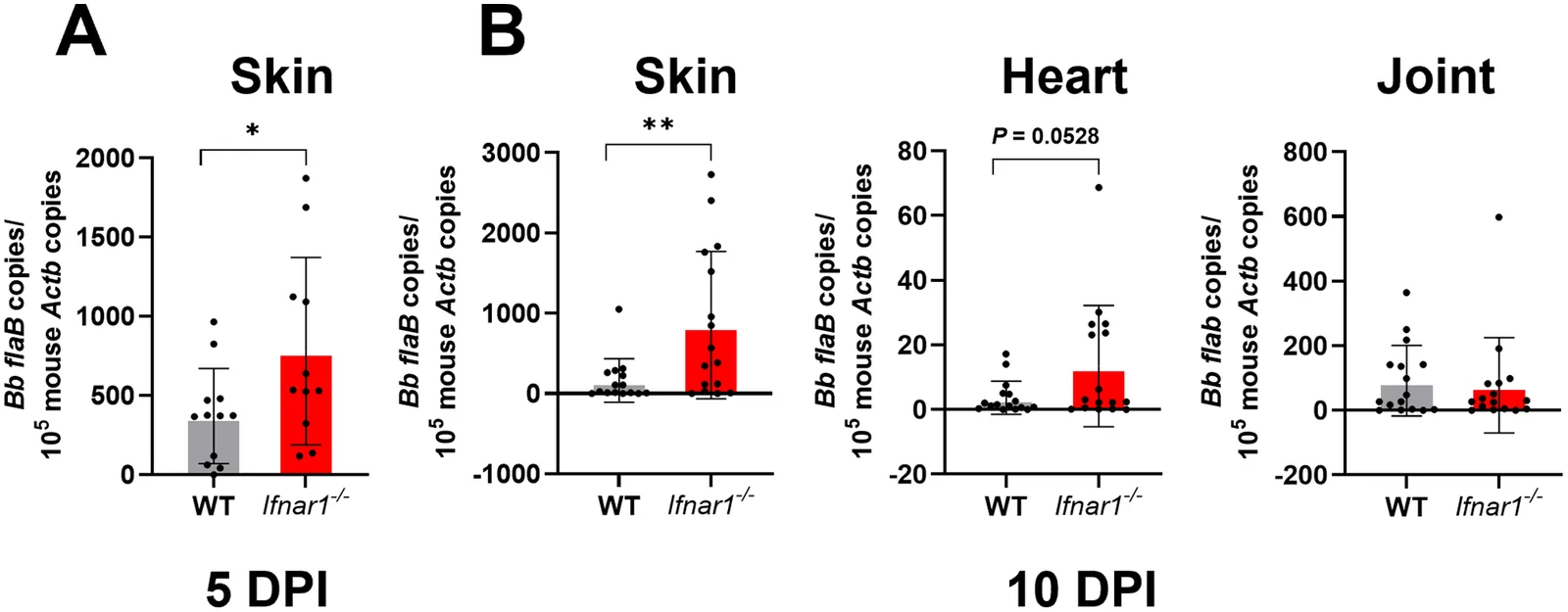
Type I IFN signaling contributes to early control of *B. burgdorferi* burden. Wild-type (WT) and *Ifnar*1*^−/^^−^* mice were intradermally (ID) inoculated with *B. burgdorferi* (1 × 10^4^ spirochetes per mouse). At 5 days post-inoculation (DPI), skin tissue from the inoculation site was harvested **(A)**, while at 10 DPI, skin (site of inoculation), heart, and joint tissues were harvested **(B)**; DNA was isolated, and *B. burgdorferi* burden was quantified by quantitative PCR (qPCR). *B. burgdorferi flaB* copies were normalized to mouse *β-actin*. Data are presented as mean ± SD (n = 11–17 mice per group) from three independent experiments. Statistical significance was determined using a two-tailed unpaired Student’s *t*-test. *, *P* < 0.05; **, *P* < 0.01.

### Type I IFN signaling enhances macrophage phagocytosis of *B. burgdorferi in vitro*

To investigate the mechanisms by which IFN-I signaling contributes to the early control of *B. burgdorferi*, we first examined macrophage phagocytosis *in vitro*. Bone marrow-derived macrophages (BMDMs) from WT and *Ifnar1^−/^^−^* mice, and RAW 264.7 macrophages treated with control IgG or an IFNAR1-blocking antibody, were incubated with GFP-expressing *B. burgdorferi* at a multiplicity of infection (MOI) of 10 for 2.5 h, and bacterial uptake was assessed by flow cytometry and confocal microscopy. Flow cytometric analysis revealed a significant reduction in bacterial uptake by *Ifnar1^−/^^−^* BMDMs compared with WT controls, as indicated by both a decreased percentage of GFP-positive cells and reduced GFP mean fluorescence intensity (MFI) (Fig 2A). Similarly, blockade of IFNAR1 in RAW 264.7 macrophages resulted in a marked decrease in the percentage of GFP-positive cells and GFP MFI compared with isotype-treated controls (Fig 2B). Confocal microscopy further confirmed these findings, showing reduced intracellular GFP signal in *Ifnar1^−/^^−^* BMDMs and in RAW 264.7 cells treated with IFNAR1-blocking antibody (Fig 2C).

**Fig 2:**
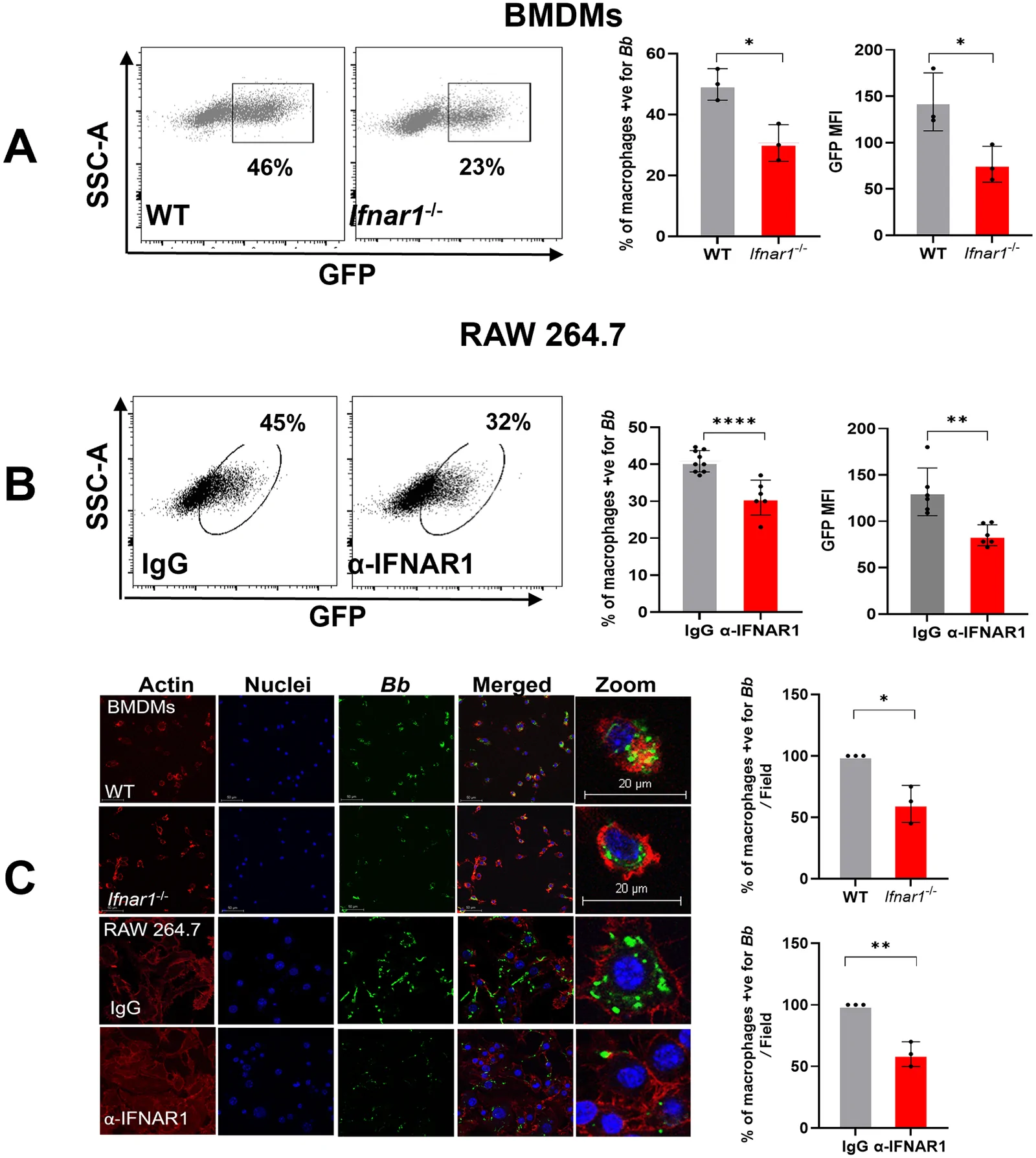
Type I IFN signaling enhances macrophage-mediated phagocytosis of *B. burgdorferi in vitro.* Bone marrow-derived macrophages (BMDMs) from wild-type (WT) and *Ifnar1^−/^^−^* mice, and RAW 264.7 macrophages treated with control IgG or an IFNAR1-blocking antibody were incubated with GFP-expressing *B. burgdorferi* at a multiplicity of infection (MOI) of 10 for 2.5 h**. (A, B)** Flow cytometric analysis of phagocytosis. Representative flow cytometry plots (SSC versus GFP) and quantification of the percentage (%) of GFP-positive cells and GFP mean fluorescence intensity (MFI) in **(A)** BMDMs and **(B)** RAW 264.7 cells. **(C)** Confocal microscopy analysis. Representative confocal images showing GFP-*B. burgdorferi* (green) within BMDMs and RAW 264.7 macrophages, with quantification of the percentage (%) of GFP-positive macrophages. Data are presented as mean ± SD from at least three independent experiments. Statistical significance was determined using a two-tailed unpaired Student’s *t*-test. *, *P* < 0.05; **, *P* < 0.01; ****, *p* < 0.0001.

To confirm bacterial internalization, confocal Z-stacks were acquired and analyzed using orthogonal views and selected optical Z-sections (S1A Fig). GFP-*B. burgdorferi* signals were detected within the cellular Z-volume of both WT and *Ifnar1^−/^^−^* BMDMs (S1A and S1B Fig). In addition, quenching of extracellular GFP fluorescence with trypan blue showed that the reduced GFP signal in *Ifnar1^−/^^−^* BMDMs persisted after exclusion of extracellular bacterial fluorescence (S1C Fig). Together, these complementary approaches confirm the intracellular localization of macrophage-associated GFP-*B. burgdorferi* and further support our conclusion that IFNAR1 deficiency impairs bacterial uptake.

To determine whether the effect of IFN-I signaling on macrophage phagocytosis was specific to *B. burgdorferi*, we performed parallel phagocytosis assays using FITC-labeled *E. coli*. Flow cytometric analysis showed comparable percentages of FITC-positive cells and FITC MFI in WT and *Ifnar1^−/^^−^* BMDMs following exposure to *E. coli* (S2A Fig). Consistent with these results, confocal microscopy revealed comparable intracellular FITC-*E. coli* signals in WT and *Ifnar1^−/^^−^* BMDMs, with no significant difference in quantified FITC fluorescence between the two groups (S2B Fig). Together, these findings indicate that the effect of IFN-I signaling on macrophage phagocytosis is not universal but bacteria-dependent, with a more pronounced effect on *B. burgdorferi* under the conditions tested.

### Type I IFN signaling promotes macrophage phagocytosis of *B. burgdorferi in vivo*

To determine whether IFN-I signaling contributes to macrophage phagocytosis of *B. burgdorferi in vivo*, WT and *Ifnar1^−/^^−^* mice were inoculated intraperitoneally (IP) with GFP-expressing *B. burgdorferi*. Peritoneal exudate cells were harvested 6 hours post-inoculation (HPI), and macrophage phagocytosis was assessed by flow cytometry*,* as described previously [37] (Fig 3A). Macrophages were identified as live CD45^+^CD11b^+^F4/80^+^ cells using a sequential gating strategy (Fig 3B). Representative flow cytometry plots (GFP versus SSC) demonstrated reduced GFP signal in peritoneal macrophages from *Ifnar1^−/^^−^* mice compared with WT controls (Fig 3C). Quantitative analysis revealed a significantly lower percentage of GFP-positive macrophages in *Ifnar1^−/^^−^* mice compared with WT mice (Fig 3D), indicating reduced bacterial uptake in the absence of IFN-I signaling.

**Fig 3:**
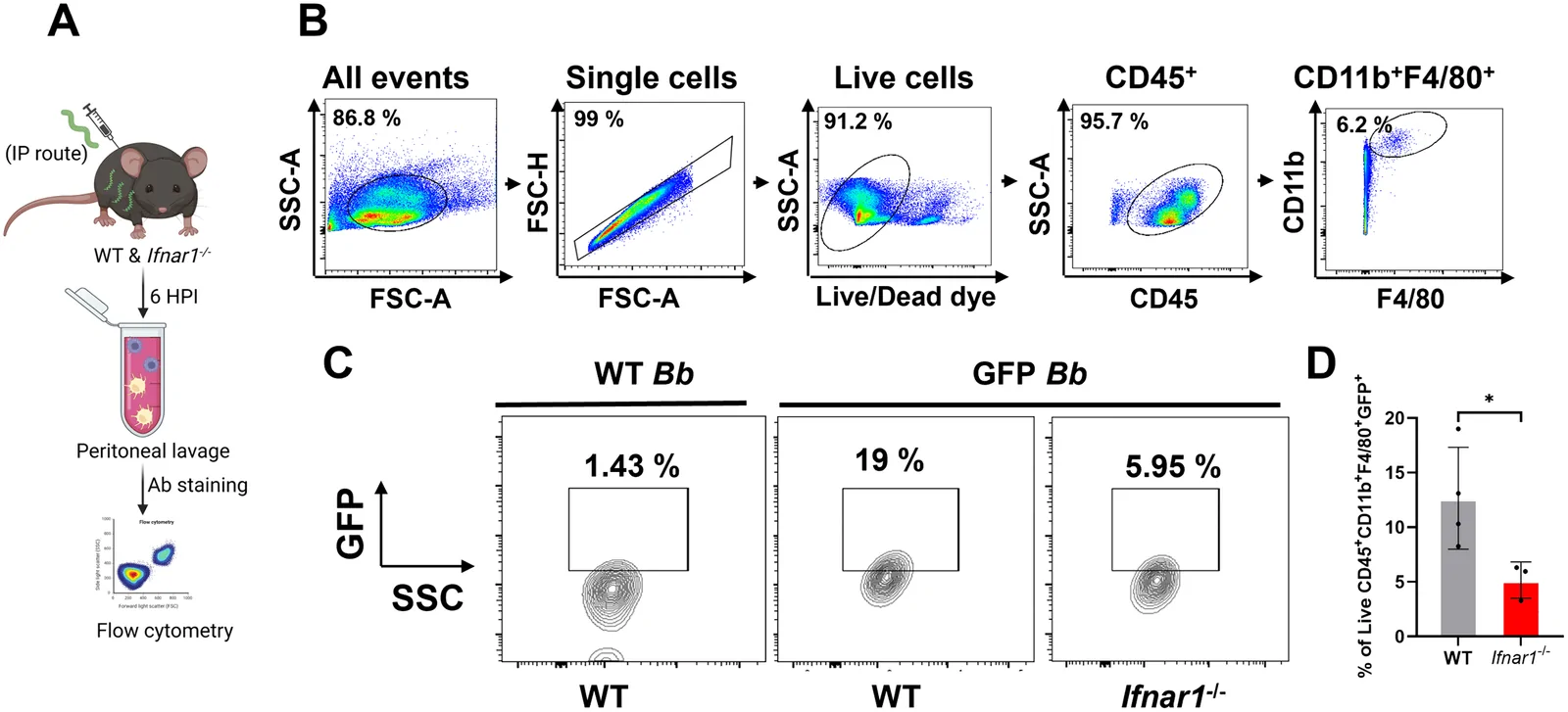
Type I IFN signaling promotes macrophage-mediated phagocytosis of *B. burgdorferi in vivo.* Wild-type (WT) and *Ifnar1^−/^^−^* mice were inoculated intraperitoneally (IP) with GFP-expressing *B. burgdorferi* (1 × 10⁷ spirochetes per mouse), or with non-fluorescent *B. burgdorferi* (as a control for autofluorescence). Peritoneal exudate cells (PECs) were harvested at 6 hours post-inoculation (HPI) and stained with a viability dye, anti-CD45, anti-CD11b, and anti-F4/80 antibodies. Phagocytosis was assessed by flow cytometry to detect the GFP signal in live CD45^+^CD11b^+^F4/80^+^ peritoneal macrophages. **(A)** Workflow illustrating the experimental design. **(B)** Flow cytometry gating strategy used to identify live peritoneal macrophages. **(C)** Representative flow cytometry plots (GFP vs SSC) showing GFP signal in peritoneal macrophages from WT and *Ifnar1^−/^^−^* mice. **(D)** Quantification of the percentage of GFP-positive peritoneal macrophages. Data are presented as mean ± SD (n = 3– 4 mice per group). Statistical significance was determined using a two-tailed unpaired Student’s *t-*test. *, *P* < 0.05.

### Type I IFN signaling promotes surface expression of phagocytic receptors involved in *B. burgdorferi* uptake

To investigate the mechanism underlying the reduced uptake of *B. burgdorferi* by *Ifnar1^−/^^−^* macrophages, we examined the surface expression of the phagocytic co-receptors CD11b and CD14 on BMDMs following exposure to *B. burgdorferi*. Previous studies have demonstrated important roles for both receptors in the recognition and phagocytosis of *B. burgdorferi* [37,38]. Flow cytometric analysis showed that, compared with WT controls, *Ifnar1^−/^^−^* BMDMs exhibited significantly lower percentages of CD11b⁺ and CD14^+^ cells, together with reduced MFI for both receptors (Fig 4A and 4B). These findings indicate that IFNAR1 signaling promotes surface expression of CD11b and CD14 and suggest a potential mechanism by which IFN-I signaling enhances macrophage recognition and uptake of *B. burgdorferi*.

**Fig 4:**
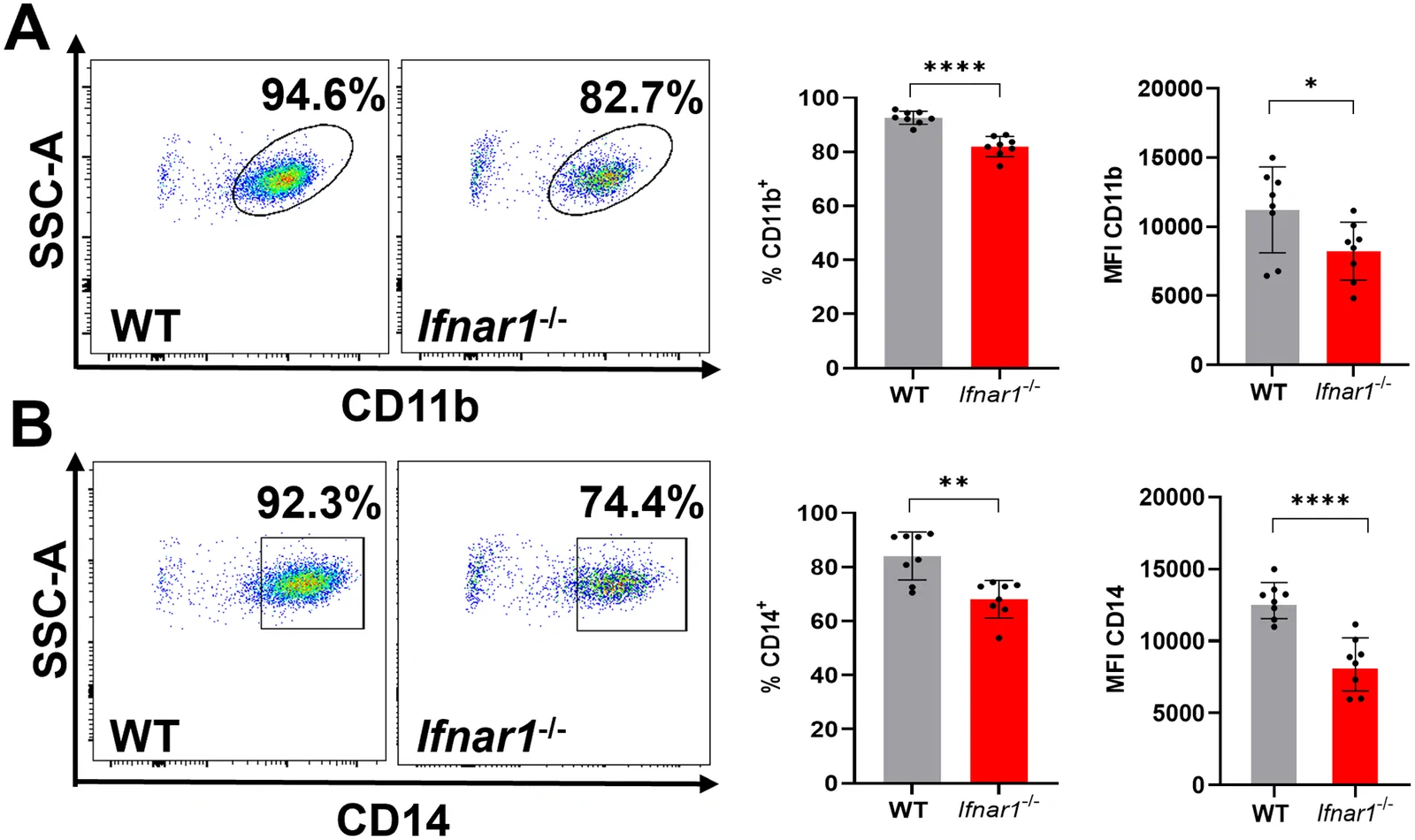
Type I IFN signaling promotes surface expression of CD11b and CD14 on macrophages. Wild-type (WT) and *Ifnar1^−/^^−^* bone marrow-derived macrophages (BMDMs) were incubated with *B. burgdorferi* at a multiplicity of infection (MOI) of 10 for 2.5 h and analyzed for cell-surface expression of CD11b and CD14 by flow cytometry. **(A)** Representative flow cytometry dot plots (left), quantification of the percentage (%) of CD11b⁺ cells (center), and mean fluorescence intensity (MFI) of CD11b (right). **(B)** Representative flow cytometry dot plots (left), quantification of the percentage of CD14^+^ cells (center), and MFI of CD14 (right). Data are presented as mean ± SD from three independent experiments. Statistical significance was determined using a two-tailed unpaired Student’s *t*-test. * *P* < 0.05, ** *P* < 0.01, **** *P* < 0.0001.

### Type I IFN signaling promotes phagosomal maturation, antimicrobial activity, and intracellular killing of *B. burgdorferi*

Following internalization, efficient killing of *B. burgdorferi* requires progressive maturation of spirochete-containing phagosomes into acidic, degradative phagolysosomes. To determine whether IFN-I signaling regulates this process, we examined early and late endolysosomal markers in WT and *Ifnar1^−/^^−^* BMDMs following incubation with GFP-*B. burgdorferi* using confocal fluorescence microscopy. Co-localization of Rab5A, a small GTPase involved in early phagosome maturation, with GFP-*B. burgdorferi*-containing phagosomes was significantly reduced in *Ifnar1^−/^^−^* BMDMs compared with WT controls (Fig 5A). Similarly, co-localization of internalized GFP-*B. burgdorferi* with lysosome-associated membrane protein 1 (LAMP1), a marker of late endosomal/lysosomal compartments, was markedly reduced in *Ifnar1^−/^^−^* macrophages (Fig 5B). To determine whether this defect was specific to *B. burgdorferi*, we also assessed LAMP1 co-localization following incubation of BMDMs with FITC-*E. coli*. In contrast to *B. burgdorferi*, no significant difference in LAMP1–*E. coli* colocalization was observed between WT and *Ifnar1^−/^^−^* macrophages (S2C Fig).

**Fig 5:**
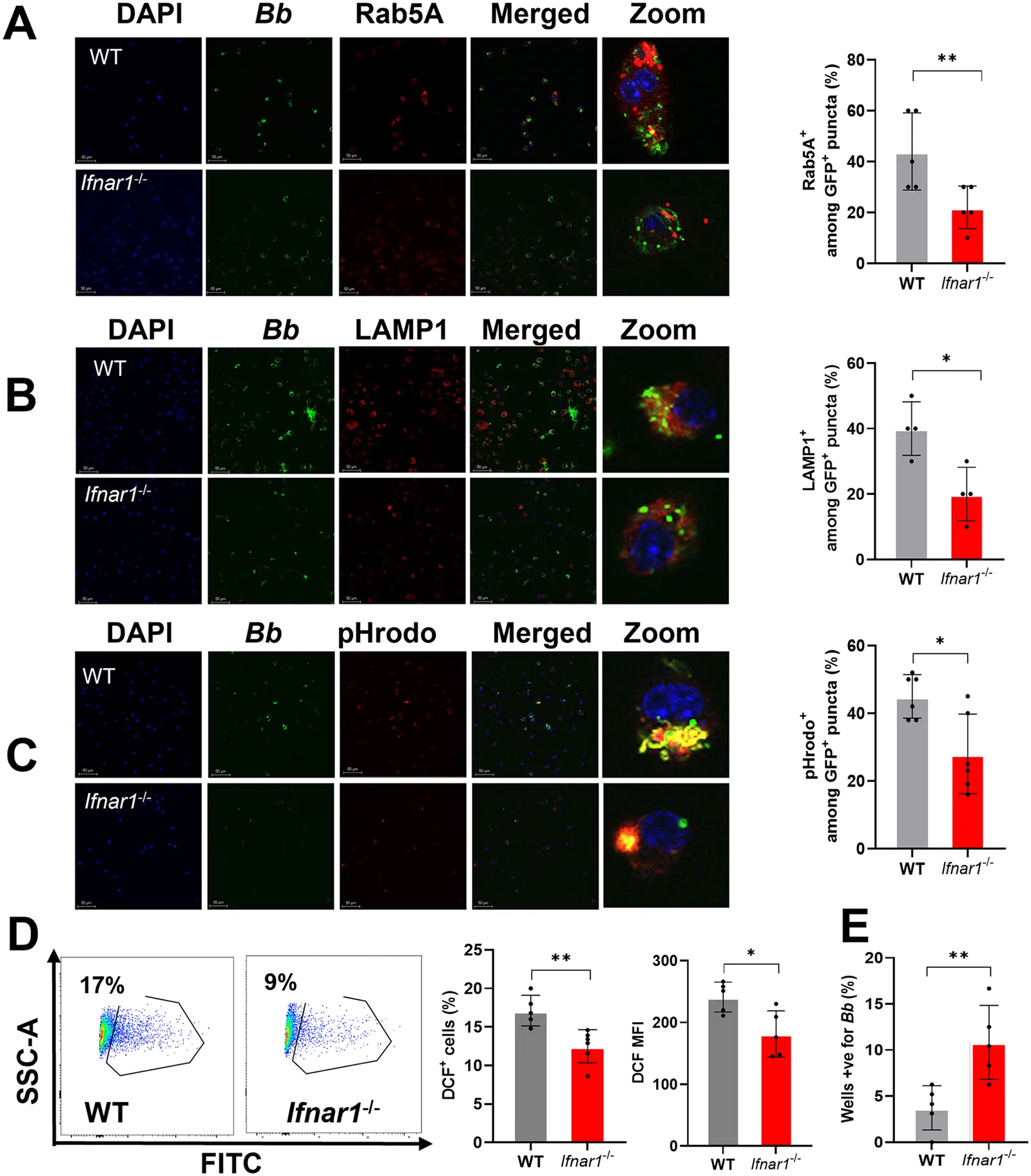
Type I IFN signaling promotes phagosome maturation, acidification, and intracellular killing of *B. burgdorferi.* WT and *Ifnar1^−/^^−^* bone marrow-derived macrophages (BMDMs) were incubated with *B. burgdorferi* at a multiplicity of infection (MOI) of 10 for 2.5 h for A, B, C, and E, and overnight for D. **(A)** Representative confocal images and quantification of Rab5A colocalization to GFP-*B. burgdorferi*-containing phagosomes. **(B)** Representative confocal images and quantification of GFP-*B. burgdorferi* colocalization with LAMP1. **(C)** Representative confocal images and quantification of pHrodo Red fluorescence associated with GFP-*B. burgdorferi* as a measure of phagosomal acidification. **(D)** Intracellular reactive oxygen species (ROS) generation measured by DCFH-DA staining and flow cytometry. Representative plots and quantification of DCF-positive cells and DCF mean fluorescence intensity (MFI) are shown. **(E)** Gentamicin protection assay. BMDMs were incubated with gentamicin (200 µg/mL) to eliminate extracellular bacteria. Following macrophage lysis, intracellular bacterial viability was determined by limiting dilution in BSK-II medium and expressed as the percentage of wells positive for *B. burgdorferi*. Data are presented as mean ± SD from two independent experiments. Statistical significance was determined using an unpaired two-tailed Student’s *t*-test. *P < 0.05, **P < 0.01.

We next determined whether impaired phagosomal maturation in *Ifnar1^−/^^−^* macrophages was accompanied by defective acidification. BMDMs were incubated with pHrodo Red-labeled GFP-*B. burgdorferi*, in which pHrodo fluorescence increases upon exposure to an acidic environment. Confocal imaging revealed a significantly lower proportion of pHrodo-positive *B. burgdorferi* in *Ifnar1^−/^^−^* BMDMs than in WT controls (Fig 5C), indicating reduced acidification of *B. burgdorferi*-containing compartments in the absence of IFN-I signaling.

To determine whether IFN-I signaling also regulates macrophage antimicrobial activity following *B. burgdorferi* uptake, we assessed reactive oxygen species (ROS) generation by measuring 2′,7′-dichlorofluorescein (DCF) fluorescence using flow cytometry. *Ifnar1^−/^^−^* BMDMs exhibited a significant reduction in the percentage of DCF-positive cells and DCF MFI compared with WT macrophages (Fig 5D).

Finally, we assessed intracellular killing of *B. burgdorferi* using a gentamicin protection assay. WT and *Ifnar1^−/^^−^* BMDMs were incubated with *B. burgdorferi*, extracellular bacteria were eliminated by gentamicin treatment, and the viability of intracellular bacteria was quantified by limiting dilution. Lysates from *Ifnar1^−/^^−^* BMDMs yielded significantly more *B. burgdorferi*-positive wells than those from WT macrophages, indicating impaired intracellular killing in the absence of IFN-I signaling (Fig 5E). Together, these findings demonstrate that IFN-I signaling promotes the maturation and acidification of *B. burgdorferi*-containing phagosomes, enhances macrophage antimicrobial activity, and facilitates intracellular killing of the spirochete.

### Exogenous IFN-β enhances macrophage phagocytosis and intracellular killing of *B. burgdorferi*

To determine whether activation of IFN-I signaling is sufficient to enhance macrophage antimicrobial function, we examined the effects of exogenous IFN-β on macrophage uptake and intracellular killing of *B. burgdorferi in vitro*. RAW 264.7 macrophages were incubated with GFP-expressing *B. burgdorferi* in the presence or absence of recombinant IFN-β, and bacterial uptake was assessed by flow cytometry and confocal microscopy (Fig 6A). Flow cytometric analysis demonstrated that IFN-β treatment significantly increased both the percentage of GFP-positive macrophages and the GFP MFI compared with untreated controls, indicating enhanced bacterial uptake (Fig 6B). Consistent with these findings, confocal microscopy revealed increased intracellular GFP signal in IFN-β-treated macrophages, with a higher proportion of GFP-positive cells relative to untreated controls (Fig 6C).

**Fig 6:**
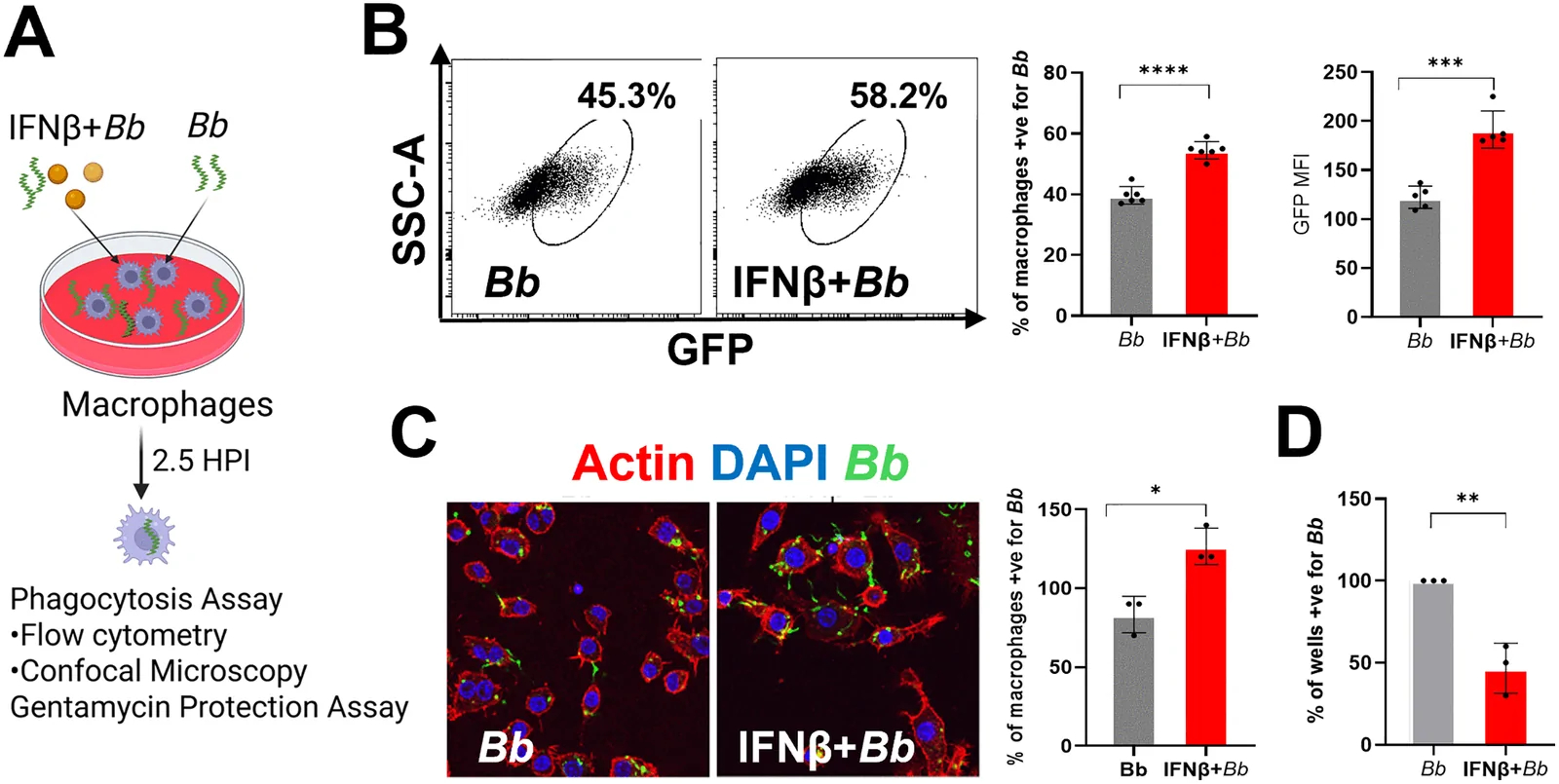
Exogenous IFN-β enhances macrophage phagocytosis and intracellular killing of *B. burgdorferi.* RAW 264.7 macrophages were incubated with GFP-expressing *B. burgdorferi* at a multiplicity of infection (MOI) of 10 for 2.5 h in the presence or absence of exogenous recombinant IFN-β (500 pg/mL). **(A)** Experimental schematic outlining IFN-β treatment, incubation of macrophages with *B. burgdorferi*, and downstream analyses. **(B)** Flow cytometric analysis of phagocytosis. Representative plots and quantification of the percentage of GFP-positive macrophages and GFP mean fluorescence intensity (MFI). **(C)** Confocal microscopy analysis. Representative images showing intracellular GFP-*B. burgdorferi* and quantification of GFP-positive macrophages. **(D)** Gentamicin protection assay assessing intracellular bacterial viability following IFN-β treatment. Data are presented as mean ± SD from three independent experiments. Statistical significance was determined using a two-tailed unpaired Student’s *t-*test. *, *P* < 0.05; **, *P* < 0.01; ***, *P* < 0.001; ****, *P* < 0.0001.

To assess whether IFN-β treatment also affected intracellular bacterial viability, we performed a gentamicin protection assay following infection. IFN-β-treated macrophages exhibited a reduced percentage of wells positive for spirochetes compared with untreated cells, indicating enhanced intracellular killing of *B. burgdorferi* (Fig 6D). Together, these results demonstrate that treatment with exogenous IFN-β is sufficient to enhance macrophage phagocytosis and intracellular killing of *B. burgdorferi in vitro*.

### Type I IFN signaling modulates inflammatory cytokine responses to *B. burgdorferi*

Because recognition and uptake of *B. burgdorferi* by macrophages are closely linked to inflammatory cytokine production, we next examined whether IFN-I signaling contributes to macrophage cytokine responses to *B. burgdorferi*. WT and *Ifnar1^−/^^−^* BMDMs were incubated with *B. burgdorferi*, and levels of IL-1β, IL-6, TNF-α, IFN-α1, and IFN-β in culture supernatants were measured at different time points. At 24 h, *Ifnar1^−/^^−^* BMDMs produced significantly lower levels of IL-1β and TNF-α than WT cells (Fig 7A). IL-6 levels were also reduced in *Ifnar1^−/^^−^* BMDMs at earlier time points but approached WT levels by 24 h. In contrast, IFN-α1 and IFN-β levels did not differ significantly between WT and *Ifnar1^−/^^−^* macrophages.

**Fig 7:**
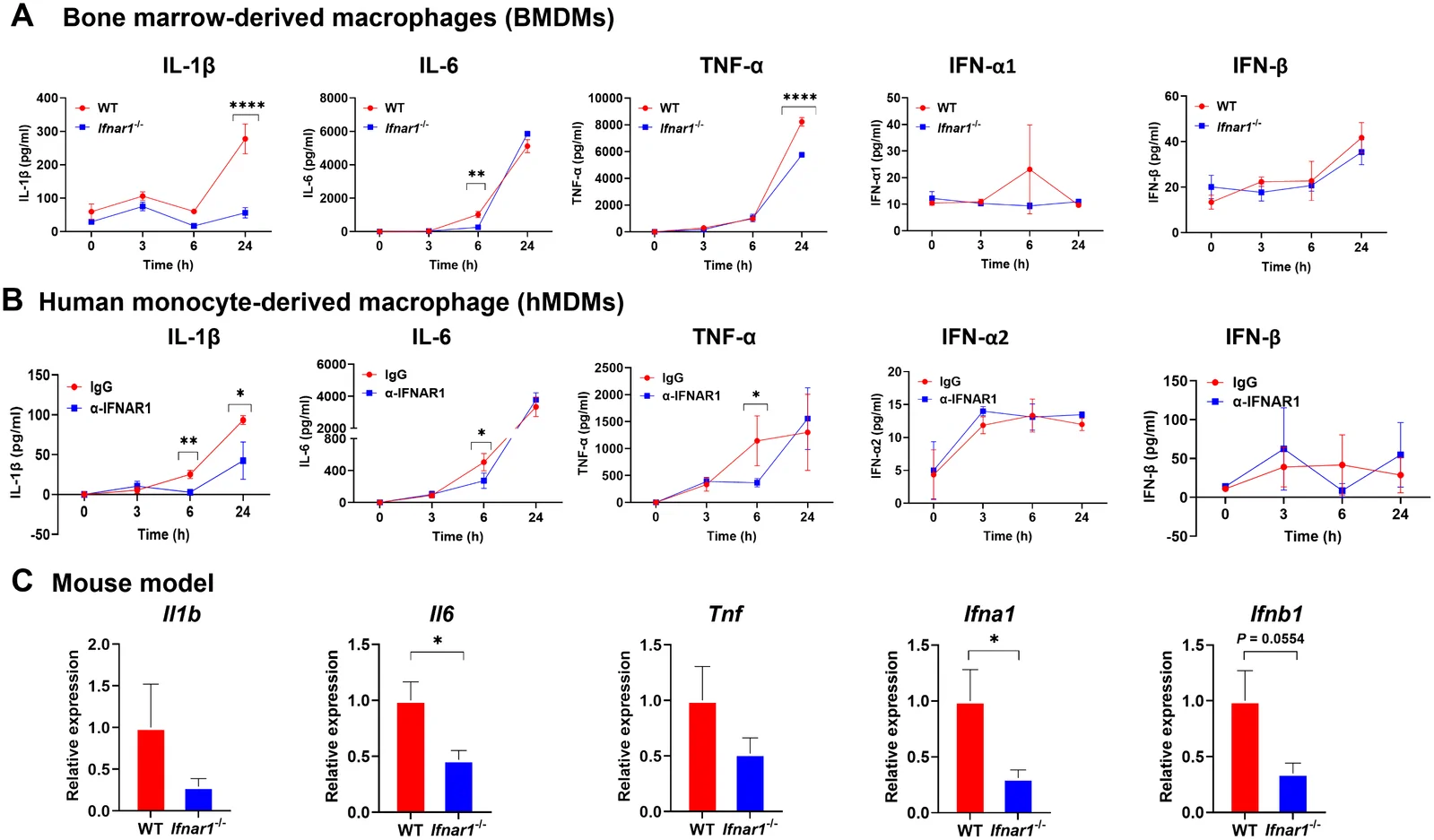
Type I IFN signaling modulates inflammatory cytokine responses to *B. burgdorferi.* **(A)** Bone marrow-derived macrophages (BMDMs) from wild-type (WT) and *Ifnar1^−/^^−^* mice and (**B**) human monocyte-derived macrophages (hMDMs) treated with an IgG control or an IFNAR1-blocking antibody were incubated with *B. burgdorferi* at a multiplicity of infection (MOI) of 10 for the indicated time points. Levels of IL-1β, IL-6, TNF-α, IFN-β, and IFN-α1 (BMDMs) or IFN-α2 (hMDMs) were measured by ELISA. **(C)** WT and *Ifnar1^−/^^−^* mice were inoculated intradermally (ID) with *B. burgdorferi* (1 × 10⁴ spirochetes per mouse). Skin tissues from the inoculation site were collected at 5 days post-inoculation (DPI), and total RNA was isolated. Transcript levels of *Il1b*, *Il6*, *Tnf*, *Ifna1*, and *Ifnb1* were quantified by quantitative RT-PCR (qRT-PCR), normalized to *β-actin,* and expressed relative to WT controls. Data are presented as mean ± SD from three independent experiments (n = 8-13 mice per group for **C**). Statistical significance was determined using a two-tailed unpaired Student’s *t-*test. *, *P* < 0.05; **, *P* < 0.01; ****, *P* < 0.0001.

To determine whether a similar effect occurs in human macrophages, human monocyte-derived macrophages (hMDMs) were incubated with *B. burgdorferi* in the presence of an isotype IgG control or an IFNAR1-blocking antibody. IFNAR1 blockade significantly reduced IL-1β levels at 6 and 24 h and reduced IL-6 and TNF-α levels at 6 h (Fig 7B). Similar to the findings in mouse BMDMs, IFN-I production, measured as IFN-α2 and IFN-β, was not significantly altered by IFNAR1 blockade.

Finally, to assess the effect of IFNAR1 signaling on inflammatory cytokine expression *in vivo*, we analyzed cytokine gene expression at the inoculation site in WT and *Ifnar1^−/^^−^* mice at 5 DPI. Compared with WT controls, *Ifnar1^−/^^−^* mice showed lower expression of the cytokine genes examined, with *Il6* significantly reduced (Fig 7C). Notably, in contrast to the *in vitro* macrophage experiments, expression of *Ifna1* and *Ifnb1* was also reduced in *Ifnar1^−/^^−^* mice, with the decrease in *Ifna1* reaching statistical significance. These findings suggest that the effect of IFNAR1 signaling on IFN-I expression differs between isolated macrophages *in vitro* and the more complex tissue environment *in vivo*. Nevertheless, the data demonstrate that IFN-I signaling modulates inflammatory cytokine responses to *B. burgdorferi* in both mouse and human macrophages and during early infection *in vivo*.

### Type I IFN signaling promotes macrophage recruitment to the site of *B. burgdorferi* inoculation

To determine whether IFN-I signaling influences innate immune cell recruitment during early *B. burgdorferi* infection, WT and *Ifnar1^−/^^−^* mice were inoculated intradermally (ID), and immune cell populations at the inoculation site were analyzed by flow cytometry at 5 DPI. Using a sequential gating strategy, macrophages were identified as live CD45^+^CD11b^+^F4/80^+^ cells, MHC-II^high^ macrophages as live CD45^+^CD11b⁺F4/80^+^I-A/I-E^high^ cells, and neutrophils as live CD45^+^CD11b⁺Ly6G⁺ cells (Fig 8A). Compared with WT mice, *Ifnar1^−/^^−^* mice exhibited significantly lower frequencies of total CD45^+^ cells, macrophages, and MHC-II^high^ macrophages (Fig 8B). In contrast, neutrophil frequencies were comparable between the two groups. These findings indicate that IFN-I signaling promotes macrophage accumulation at the cutaneous inoculation site during early *B. burgdorferi* infection, whereas neutrophil accumulation was not detectably altered at this time point.

**Fig 8:**
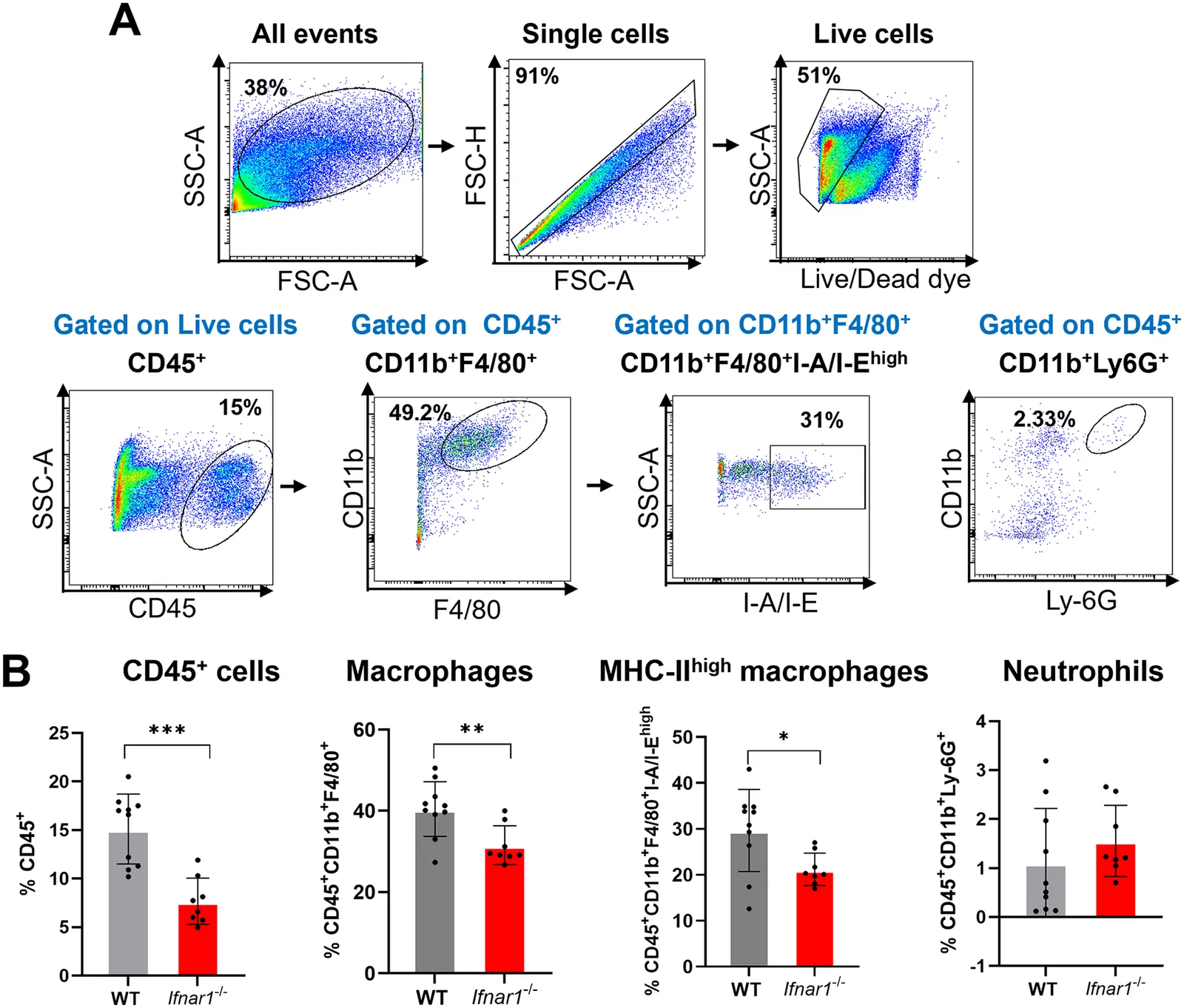
Type I IFN signaling promotes recruitment of innate immune cells during early *B. burgdorferi* infection. WT and *Ifnar1^−/^^−^* mice were inoculated intradermally (ID) with *B. burgdorferi* (1 × 10⁴ spirochetes per mouse). Skin tissues from the inoculation site were collected at 5 days post-inoculation (DPI), processed into single-cell suspensions, and analyzed by flow cytometry. **(A)** Representative gating strategy used to identify CD45+, macrophages (live CD45^+^CD11b^+^F4/80^+^), MHC-II^high^ macrophages (live CD45^+^CD11b⁺F4/80^+^I-A/I-E^high^), and neutrophils (live CD45^+^CD11b^+^Ly6G^+^). **(B)** Quantification of CD45+, macrophages, MHC-II^high^ macrophages, and neutrophils, respectively. Data are presented as mean ± SD (n = 8–10 mice per group) from two independent experiments. Statistical significance was determined using a two-tailed unpaired Student’s *t*-test. *P < 0.05; **P < 0.01.

To complement the findings from the cutaneous inoculation site and further assess the role of IFN-I signaling in acute innate immune cell recruitment, we examined macrophage and neutrophil responses in an intraperitoneal (IP) *B. burgdorferi* challenge model. Consistent with the reduced macrophage accumulation observed in the skin, *Ifnar1^−/^^−^* mice exhibited reduced macrophage recruitment to the peritoneal cavity following *B. burgdorferi* challenge. In contrast to the ID model, however, neutrophil recruitment was also significantly reduced in *Ifnar1^−/^^−^* mice in the IP model (S3 Fig). Together, these findings support a role for IFN-I signaling in promoting macrophage accumulation during early *B. burgdorferi* infection, while its effect on neutrophil recruitment appears to vary with tissue context and/or the timing of the response.

### STING signaling contributes to type I IFN expression during *B. burgdorferi* infection *in vivo*

Having established a role for IFN-I signaling in the early control of *B. burgdorferi* burden, we next examined whether the cGAS-STING pathway contributes to IFN-I induction during infection *in vivo*. We and others previously identified cGAS-STING as an important pathway contributing to *B. burgdorferi*-induced IFN-I responses *in vitro* [24,25]. To assess its contribution *in vivo*, we measured *Ifna1* and *Ifnb1* expression in infected skin from WT, *Cgas^−/^^−^*, and *Sting1*^-/-^mice by quantitative RT-PCR (qRT-PCR) at 5 DPI.

Compared with WT mice, *Sting1*^-/-^ mice exhibited reduced *Ifna1* expression, although the difference did not reach statistical significance (*P* = 0.0543) (Fig 9, left), whereas *Ifnb1* expression was significantly reduced (Fig 9, right). Expression of both *Ifna1* and *Ifnb1* was also lower in *Cgas^−/^^−^* mice than in WT mice, although these differences were not statistically significant. Together, these findings support a role for STING signaling in promoting early IFN-I expression during *B. burgdorferi* infection *in vivo*.

**Fig 9:**
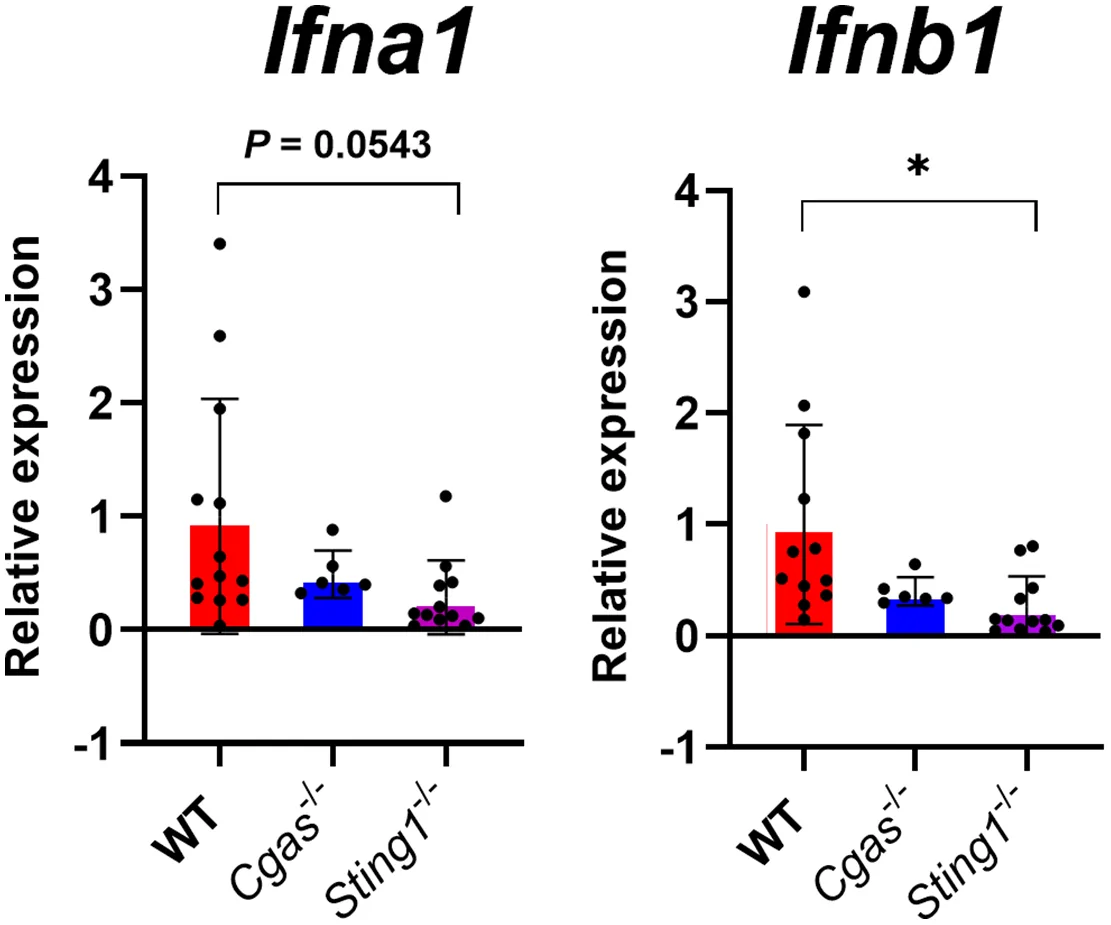
STING signaling contributes to type I interferon expression during *B. burgdorferi* infection *in vivo.* Wild-type (WT), *Cgas*^−/−,^ and *Sting1*^−/−^ mice were inoculated intradermally (ID) with *B. burgdorferi* (1 × 10⁴ per mouse). Skin tissues were collected from the inoculation site at 5 days post-inoculation (DPI), and RNA was isolated. Relative expression of *Ifna1* and *Ifnb1* was determined by quantitative RT-PCR (qRT-PCR). Gene expression was normalized to *β-actin* and expressed relative to the mean expression in WT mice. Data are presented as mean ± SD (n = 6–12 mice per group) from at least two independent experiments. Statistical significance was determined using one-way ANOVA followed by Sidak’s multiple-comparisons test. *P < 0.05.

### Pharmacological activation of IFN-I signaling enhances *B. burgdorferi* clearance *in vitro* and *in vivo*

Having shown that IFN-I signaling promotes macrophage uptake and intracellular killing of *B. burgdorferi* and contributes to early bacterial control *in vivo*, we next asked whether pharmacological activation of IFN-I signaling could enhance host control of infection. Because bacterial cyclic di-adenosine monophosphate (c-di-AMP) is a STING agonist that induces IFN-I responses, we used c-di-AMP to augment IFN-I response *in vitro* and *in vivo*. RAW 264.7 macrophages were pretreated with c-di-AMP and subsequently incubated with GFP-*B. burgdorferi* at low and high MOIs . c-di-AMP pretreatment significantly enhanced macrophage uptake of *B. burgdorferi*, as indicated by increased percentages of GFP-positive cells and higher GFP MFI compared with untreated controls (Fig 10A). Gentamicin protection assays further showed significantly reduced intracellular bacterial viability in c-di-AMP–treated macrophages, consistent with enhanced intracellular killing (Fig 10B).

**Fig 10:**
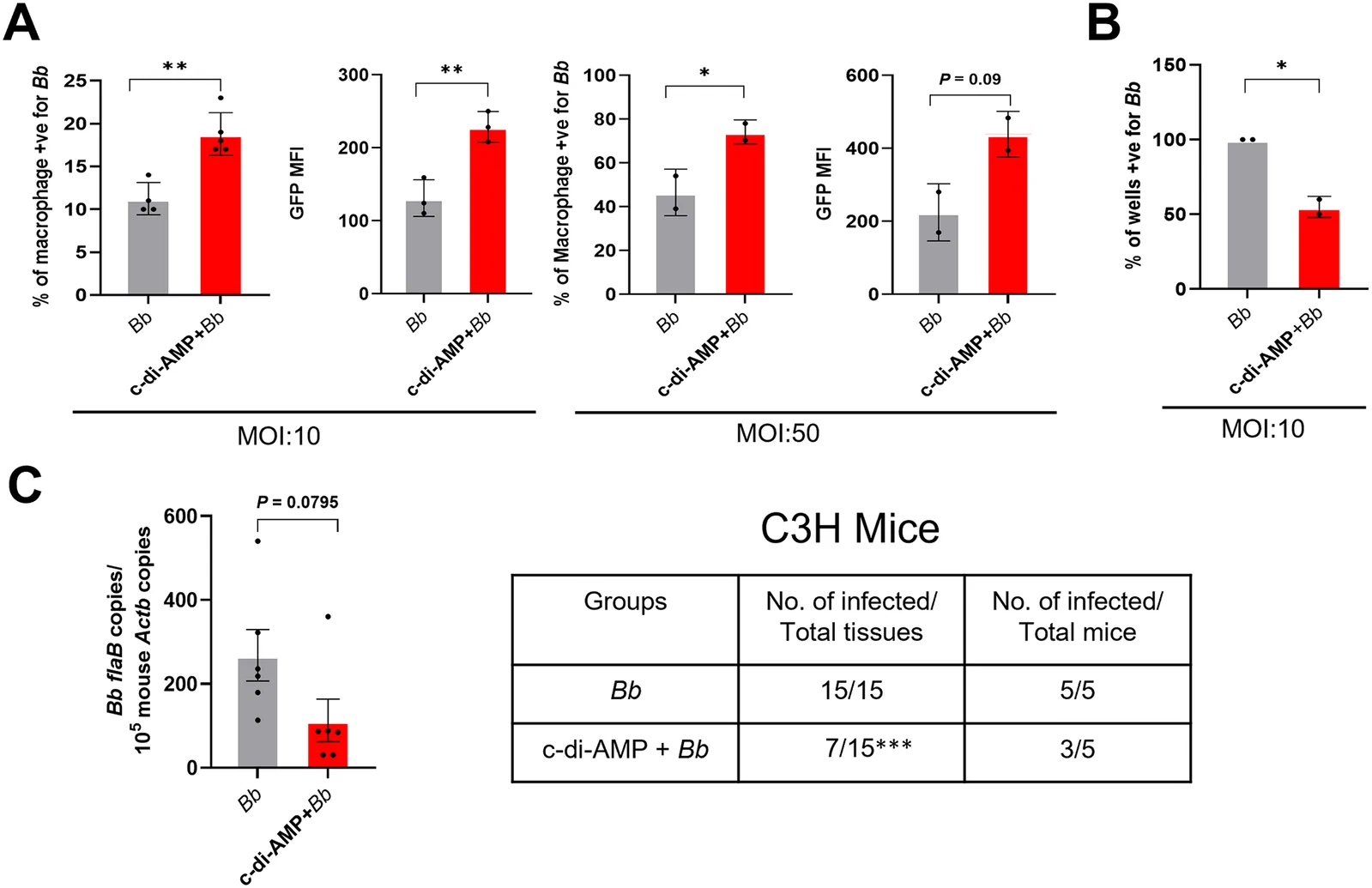
Pharmacological activation of IFN-I signaling enhances *B. burgdorferi* clearance *in vitro* and *in vivo.* (**A**) *In vitro* macrophage assays. RAW 264.7 macrophages were pretreated with c-di-AMP before incubation with GFP-expressing *B. burgdorferi* at low and high multiplicities of infection (MOI). Phagocytosis was assessed by flow cytometry to determine the percentage of GFP-positive cells and GFP mean fluorescence intensity (MFI). (**B**) Gentamicin protection assay. To assess intracellular spirochete viability, RAW 264.7 macrophages were pretreated with c-di-AMP and subsequently incubated with *B. burgdorferi* for 2.5 h, followed by treatment with gentamicin (200 µg/mL) to eliminate extracellular bacteria. Following washing and lysis, cell lysates were diluted in BSK-II medium and plated in 96-well plates. Intracellular bacterial viability was assessed by limiting dilution, and data are presented as percentages of *B. burgdorferi-*positive wells. (**C**) *In vivo* bacterial clearance. C3H/HeN mice were pretreated intradermally (ID) with c-di-AMP before inoculation with *B. burgdorferi* (1 × 10^4^ spirochetes per mouse). Spirochetal burden at the inoculation site was quantified by qPCR at 5 days post-inoculation (DPI) (**left**), and dissemination was assessed by the frequency of culture-positive tissues at 10 DPI (**right**). Data are presented as mean ± SD of two independent experiments (n = 5-6 mice per group). Statistical significance was determined using a two-tailed unpaired Student’s *t-*test for **(A)**, **(B),** and **(C, left)** (*, *P* < 0.05; **, *P* < 0.01) and a two-tailed Fisher’s exact test for **(C, right)** (***, *P* < 0.001).

We next determined whether c-di-AMP treatment could enhance control of *B. burgdorferi in vivo*. C3H/HeN mice were pretreated intradermally with c-di-AMP prior to inoculation with *B. burgdorferi*. qPCR analysis of skin at the inoculation site showed a trend toward reduced bacterial burden in c-di-AMP–treated mice at 5 DPI (Fig 10C, left). Notably, culture analysis at 10 DPI revealed a significantly lower frequency of culture-positive tissues in c-di-AMP–treated mice than in control mice (Fig 10C, right). Together, these findings demonstrate that pharmacological activation of IFN-I signaling with c-di-AMP enhances macrophage uptake and intracellular killing of *B. burgdorferi in vitro* and improves bacterial control during early infection *in vivo*.

## Discussion

The prevailing view of type I interferon (IFN-I) signaling during *B. burgdorferi* infection emphasizes its role in driving pathogenic outcomes, particularly the development and severity of Lyme arthritis, while considering it largely dispensable for host defense [23,28–31]. This perspective is based primarily on murine studies in which IFNAR1 deficiency reduces joint pathology without significantly affecting spirochetal burdens at four weeks post-infection [30]. Here, by focusing on the earliest phase of infection, we identify a previously underappreciated protective function of IFN-I. IFNAR1 deficiency resulted in increased *B. burgdorferi* burden at the cutaneous inoculation site as early as 5 DPI and a more pronounced difference by 10 DPI, together with a trend toward increased burden in the heart. Mechanistically, IFN-I signaling enhanced macrophage uptake of *B. burgdorferi*, phagosomal maturation and acidification, antimicrobial activity, intracellular killing, inflammatory cytokine responses, and macrophage accumulation at the inoculation site. Notably, robust IFN-I signatures have also been documented in human erythema migrans lesions and early disseminated disease [33–35]. These findings support a temporally distinct role for IFN-I during *B. burgdorferi* infection: an early protective response that promotes innate bacterial control, and a later contribution to inflammatory pathology.

A major mechanistic finding of this study is that IFN-I signaling promotes macrophage-mediated recognition, uptake, and intracellular killing of *B. burgdorferi*. Resistance to phagocytic clearance is an important determinant of *B. burgdorferi* infectivity [26,36,37,39–44]. Phagocytes recognize spirochetes through receptors including TLR2, complement receptor 3 (CR3), and scavenger receptors, activating MyD88-dependent and -independent pathways that promote bacterial uptake and degradation [26,36,37,41–44]. While effective phagocytosis reduces bacterial burden and initiates proinflammatory cytokine production [45], impaired clearance permits *B. burgdorferi* to disseminate and persist [37,40]. Conversely, *B. burgdorferi* has evolved mechanisms to evade phagocytic elimination, and its infectivity correlates with resistance to phagocyte-mediated killing [39,46]. In this study, we show that IFNAR1-deficient macrophages display impaired spirochetal uptake both *in vitro* and *in vivo*, accompanied by impaired phagosomal processing and reduced intracellular killing (Figs 2, 3, and 5). These defects are consistent with the significantly higher *B. burgdorferi* burden at the site of inoculation and a trend toward increased burden in the heart (Fig 1). Our finding that IFNAR1-deficient macrophages exhibited reduced surface expression of CD11b and CD14 (Fig 4), provides a potential mechanism for their impaired uptake of *B. burgdorferi*. Interestingly, *B. burgdorferi* linear plasmid lp36 has been shown to be required for both optimal IFN-I induction and efficient adhesion and internalization, further suggesting a functional connection between IFN-I signaling and phagocytic recognition [47].

Following internalization, IFNAR1 deficiency also impaired recruitment of Rab5A and LAMP1 to spirochete-containing compartments, reduced phagosomal acidification and ROS generation, and decreased intracellular killing (Fig 5). Notably, comparable uptake of *E. coli* (S2A and S2B Fig) and LAMP1 colocalization with *E. coli* in WT and *Ifnar1^−/^^−^* macrophages (S2C Fig) suggest that IFNAR1 deficiency does not cause a generalized defect in bacterial phagocytosis or phagosomal maturation but has a more pronounced effect on the macrophage response to *B. burgdorferi*. In addition, IFN-I signaling supports optimal pro-inflammatory cytokine responses (Fig 7), further amplifying innate immune responses that contribute to early containment of infection. Together, these findings indicate that IFN-I promotes bacterial clearance at multiple steps, from recognition and uptake to phagosomal processing and antimicrobial killing.

Beyond macrophage-intrinsic antimicrobial functions, IFN-I also contributes to macrophage accumulation at the inoculation site during early infection. *Ifnar1*^-/-^ mice exhibited reduced recruitment of macrophages in the skin at 5 DPI, whereas neutrophil frequencies were unchanged (Fig 8). Consistent with the macrophage phenotype observed in the skin, macrophage recruitment was also reduced in *Ifnar1^−/^^−^* mice following intraperitoneal challenge; however, in contrast to the skin, neutrophil recruitment was also reduced in this model (S3 Fig). Previous studies have shown that impaired macrophage or neutrophil recruitment is associated with increased spirochetal burden [23,40,48–53] whereas enhanced neutrophil chemotaxis markedly attenuates infectivity [54]. In fact, tick saliva actively compromises neutrophil function to facilitate initial infection and dissemination [55,56]. Together, these observations suggest that IFN-I contributes to early innate immune cell recruitment, with its effects varying according to cell type, tissue context, and/or timing of the response.

What are the cellular sources of IFN-I during early *B. burgdorferi* infection? Although the present study did not directly examine the cellular source of IFN-I, our *in vitro* findings demonstrate that macrophages produce IFN-I in response to *B. burgdorferi*. Previous studies demonstrated that IFN-I response to *B. burgdorferi* infection occurs independently of B and T lymphocytes, indicating innate immune cells as the primary initiators of this response [23]. Subsequent studies further identified resident myeloid cells as initiators of local IFN-I response, whereas endothelial cells and fibroblasts function as amplifiers of this response within infected tissue [30]. Together, these findings support a model in which multiple cell types, including both myeloid and non-myeloid cells, contribute to the IFN-I response upon infection with *B. burgdorferi in vivo*.

Multiple innate sensing pathways likely contribute to IFN-I induction during *B. burgdorferi* infection. Petnicki-Ocwieja et al. reported that TLR2 can contribute to IFN-I induction through TRIF and MyD88 adaptors [57]. In contrast, other studies have shown that *B. burgdorferi*-induced IFN-I response can also occur independently of TLR2, TLR4, MyD88, and TRIF [20,22,57]. More recently, we and others identified cGAS–STING as an important pathway contributing to *B. burgdorferi*-induced IFN-I responses in macrophages *in vitro* [24,25]. Here we extend these observations *in vivo,* showing that *Ifnb1* expression was significantly reduced in *Sting1^−/^^−^* mice as compared with WT mice (Fig 9), while *Ifna1* expression was also reduced but did not reach statistical significance. Reduced expression of both *Ifna1* and *Ifnb1* was also observed in *Cgas^−/^^−^* mice, although these differences were not statistically significant (Fig 9). The more modest phenotype in *Cgas^−/^^−^* mice raises the possibility that additional signals upstream of STING contribute to its activation during *B. burgdorferi* infection. Nevertheless, these findings indicate that STING contributes to early IFN-I expression during *B. burgdorferi* infection *in vivo*. This notion is further supported by the promising finding that treatment with c-di-AMP, a known agonist of STING-dependent IFN-I signaling, enhanced macrophage phagocytosis and intracellular killing *in vitro* and improved bacterial control *in vivo*, as reflected by a trend toward reduced bacterial burden at the inoculation site and a significantly lower frequency of culture-positive tissues (Fig 10). Because c-di-AMP is a relatively weak STING agonist, it will be of interest to determine whether more potent STING agonists can further enhance bacterial control in future studies.

In the present study, the effect of IFN-I signaling on early *B. burgdorferi* control was most pronounced in the skin at the inoculation site. *Ifnar1^−/^^−^* mice also exhibited a 3.6-fold increase in spirochetal burden in the heart, although this difference did not reach statistical significance. However, no appreciable difference was observed in joint tissues (Fig 1). Previous studies examining the effect of IFNAR1 blockade and deficiency in mice focused primarily on joint-associated disease and reported little or no effect of impaired IFN-I signaling on spirochetal burden [23,29]. Importantly, these studies did not examine bacterial control during the early phase of infection or specifically at the cutaneous inoculation site. Thus, rather than conflicting with previous findings, our results reveal an early, tissue-specific role for IFN-I signaling that was not addressed in earlier studies. Indeed, our observation that joint burdens were comparable between WT and *Ifnar1^−/^^−^* mice is consistent with previous reports. Why IFNAR1 deficiency has a pronounced effect on bacterial burden in the skin but not in the joints remains unclear. One possibility is that the joint represents a distinct tissue microenvironment in which local immune mechanisms may compensate for the loss of IFN-I signaling and thereby limit its impact on spirochetal burden.

A related study by Farris et al. reported no apparent differences in infection kinetics or tissue colonization between WT and *Cgas^−/^^−^* or *Sting1^−/^^−^* mice using bioluminescence imaging [24], which may appear inconsistent with the increased bacterial burden observed in *Ifnar1^−/^^−^* mice in our study. Several differences may account for this discrepancy. First, Farris et al. used a higher infectious inoculum (10^5^ spirochetes per mouse), whereas we used 10^4^ spirochetes per mouse; a higher inoculum could potentially reduce the ability to detect more subtle differences in early host control. Second, our study quantified bacterial burdens in individual tissues by qPCR, which may provide greater sensitivity for detecting tissue-specific differences than whole-animal bioluminescence imaging. Most importantly, IFNAR1 deficiency is expected to have a broader effect than loss of cGAS or STING because *B. burgdorferi* can induce IFN-I through multiple innate sensing pathways [19,20,22,24,25,57]. Consistent with this concept, our data show that loss of cGAS or STING reduces but does not abolish IFN-I expression during infection (Fig 9). Thus, whereas disruption of an individual upstream sensing pathway may leave substantial IFN-I activity intact, loss of IFNAR1 eliminates cellular responses to IFN-I regardless of the pathway responsible for their induction.

Spirochetal burden in skin tissues is critical for spirochetal acquisition by feeding *Ixodes* ticks and completion of its enzootic cycle. Milovic et al. recently reported that the white-footed mouse, *Peromyscus leucopus*, a natural reservoir of *B. burgdorferi*, has a highly attenuated IFN-I response compared with laboratory mice and other rodents [58]. Our finding that loss of IFN-I signaling in *Mus musculus* leads to increased *B. burgdorferi* burdens in the skin suggests that the attenuated IFN-I response observed in the natural reservoir host *Peromyscus leucopus* may contribute to its ability to sustain long-term cutaneous infection, thereby facilitating efficient transmission to *Ixodes* tick vectors. Interestingly, BBA57, a mammalian infection-associated *B. burgdorferi* surface lipoprotein that is positively regulated by the Rrp2–RpoN–RpoS pathway and is induced during tick feeding [59–61], has been shown to influence host IFN-I-associated responses in the skin [62]. This observation suggests that *B. burgdorferi* may have evolved strategies, including the use of BBA57, to modulate early IFN-I-associated host defenses during establishment of mammalian infection. Future studies examining early spirochetal control by BBA57-deficient bacteria, particularly in *Ifnar1^−/^^−^* mice, will help determine whether the BBA57 phenotype is mediated, at least in part, through IFN-I-dependent responses.

There are several limitations in this study. First, needle inoculation does not fully reproduce natural tick transmission, in which spirochetes are delivered gradually together with immunomodulatory salivary components and undergo changes in surface protein expression. Studies using tick-mediated infection will therefore be important to determine whether the protective role of IFN-I observed in this study similarly operates during natural transmission. Second, although our mechanistic studies focused on macrophages, IFNAR1 signaling in other cell types, including stromal cells and additional innate immune cells, may contribute to host protection. Further investigation using cell type-specific IFNAR1 knockout mice would provide important information regarding the relative contribution of macrophages and other cell populations to IFNAR1-mediated protection *in vivo*.

In summary, our findings support a model in which IFN-I signaling exerts temporally distinct effects during *B. burgdorferi* infection (Fig 11). During the earliest phase of infection, IFN-I acts as an amplifier of innate immunity, promoting phagocytic receptor expression and bacterial uptake, facilitating phagosome maturation and acidification, enhancing antimicrobial effector responses and intracellular killing, augmenting pro-inflammatory cytokine production, and facilitating immune cell recruitment under the experimental conditions examined, thereby contributing to early bacterial control. In contrast, sustained or excessive IFN-I signaling at later stages contributes to inflammatory pathology, including Lyme arthritis [23]. This temporally integrated framework reconciles previously perceived pathogenic roles of IFN-I with its important early protective function. Collectively, these findings suggest that precisely timed modulation of IFN-I pathways may enhance early host defense while limiting downstream immunopathology.

**Fig 11:**
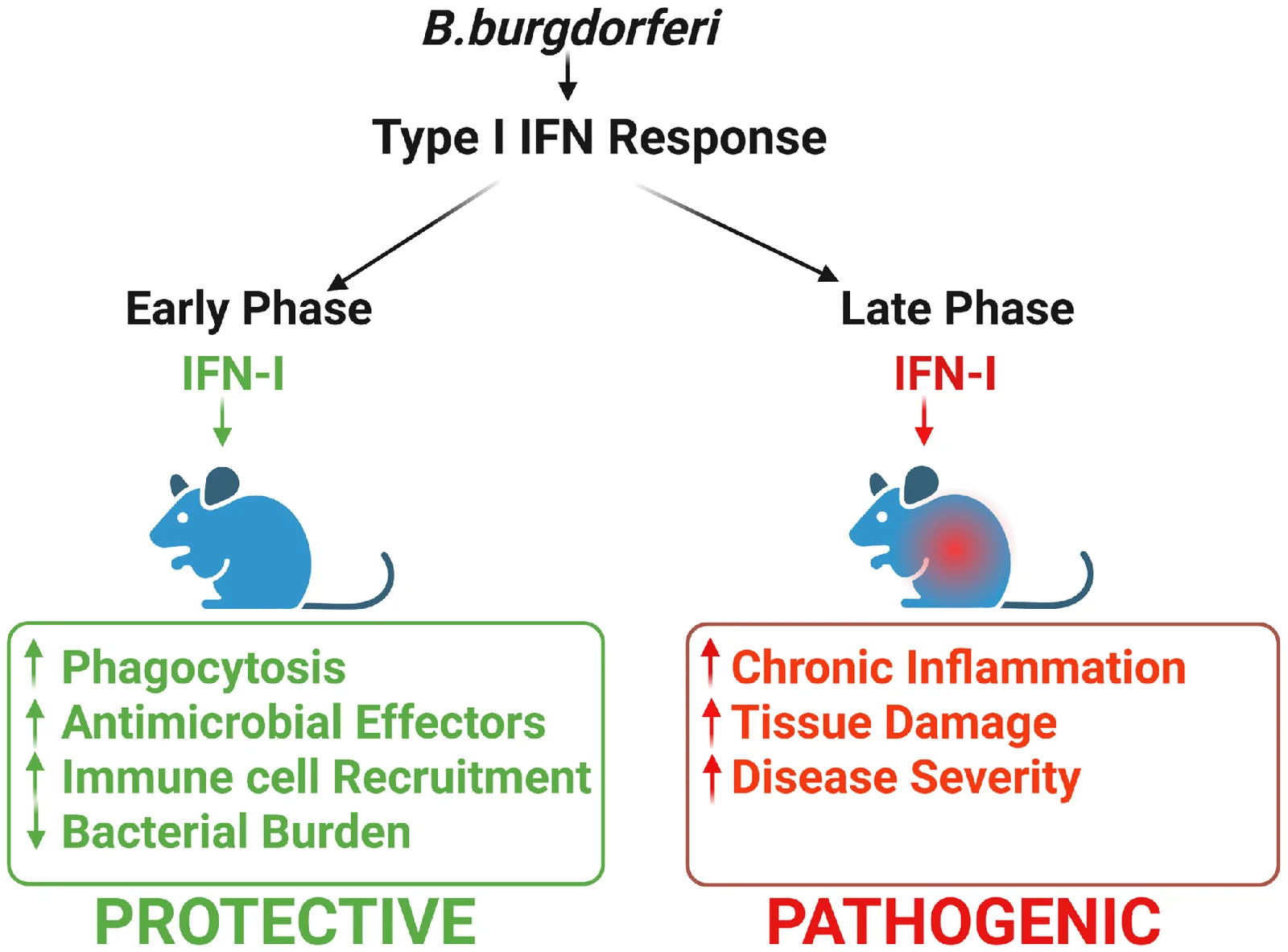
Proposed model illustrating dual roles of type I IFN signaling during *B. burgdorferi* infection. Early during infection, *B. burgdorferi* induces IFN-I signaling, which promotes protective host defense by enhancing macrophage phagocytosis, facilitating intracellular killing through lysosomal targeting, inducing pro-inflammatory cytokine production, and supporting recruitment of innate immune cells to the site of needle inoculation. These coordinated responses control spirochetal loads at the inoculation site. During later stages of infection, sustained or dysregulated IFN-I signaling contributes to inflammatory pathology, including the development of Lyme arthritis. Together, this model highlights the context- and time-dependent nature of IFN-I signaling during *B. burgdorferi* infection, in which early IFN-I responses are protective, whereas prolonged IFN-I activity contributes to disease pathology.

## Materials and methods

### Ethics statement

All animal experiments were approved by the Indiana University School of Medicine’s Institutional Animal Care and Use Committee under protocol number 23155 and were performed following the institutional guidelines for animal use. Mice were housed at the Laboratory Animal Research Centre (LARC) facility.

### Mice

Wild-type (WT) C3H/HeN, and C57BL/6 mice, and *Cgas*^-/-^, *Sting1*^-/-^ and *Ifnar1^−/^^−^* mice (on a C57BL/6 background), were purchased from the Jackson Laboratory (Bar Harbor, ME) and bred in-house at the Indiana University LARC facility. Both male and female mice aged 4–8 weeks were used for all the experiments. Mice were housed under specific pathogen-free conditions in filter-top cages with a 12-h light/dark cycle and provided food and water *ad libitum*.

### *B. burgdorferi* strains and culture conditions

The virulent *B. burgdorferi* strain 5A4NP1 was used for all the infection studies. A GFP-expressing strain was generated by electroporation of the pTM61-GFP plasmid, which drives GFP expression under the control of the *flaB* promoter. *B. burgdorferi* strains were cultured in complete Barbour-Stoenner-Kelly II (BSK-II) medium supplemented with 6% normal rabbit serum at 37°C with appropriate antibiotics. Antibiotics were used at the following concentrations: gentamicin at 50 µg/mL, and kanamycin at 100 µg/mL. Cultures were visually monitored for spirochete motility and density by dark-field microscopy.

### Cell lines

Murine macrophage cells RAW 264.7 (ATCC, Manassas, VA) were cultured in Dulbecco’s Modified Eagle medium (DMEM) with L-glutamine supplemented with 10% heat-inactivated fetal bovine serum (FBS) and 1% penicillin-streptomycin. L929 mouse fibroblasts were maintained in DMEM supplemented with 10% FBS using standard culture conditions.

### Preparation of bone marrow-derived macrophages (BMDMs)

Primary BMDMs were generated from femurs of 4–8-week-old WT and *Ifnar1^−/^^−^* mice as described earlier [63]. In brief, bone marrow cells were flushed from mouse femurs with sterile Roswell Park Memorial Institute (RPMI) 1640 medium and cultured for 7 days in RPMI 1640 supplemented with 30% L929 cell-conditioned medium, 20% FBS, and 1% penicillin-streptomycin. Differentiated macrophages were harvested and seeded into tissue culture plates for subsequent experiments.

### Generation of human monocyte-derived macrophages (hMDMs)

hMDMs were generated based on a previously described protocol with minor modifications [64]. Briefly, cryopreserved human peripheral blood mononuclear cells (PBMCs) from three healthy donors were purchased from iXCells (San Diego, CA, USA). PBMCs were thawed according to the manufacturer’s instructions, washed, and cultured in complete RPMI 1640 medium supplemented with 10% FBS and 1% penicillin-streptomycin. Monocytes were enriched by plastic adherence, with non-adherent cells removed by gentle washing after 2 h of incubation. The adherent monocytes were differentiated into macrophages by culture for 7 days in complete RPMI 1640 medium supplemented with recombinant human macrophage colony-stimulating factor (Human M-CSF; BioLegend, 574802) at a final concentration of 50 ng/mL. Fresh medium containing M-CSF was replenished every 2–3 days. Differentiated hMDMs were subsequently incubated with *B. burgdorferi* for cytokine analysis.

### Mouse infection and *B. burgdorferi* burden determination by qPCR

WT and *Ifnar1^−/^^−^* mice were inoculated intradermally with *B. burgdorferi* (1 x 10^4^ spirochetes per mouse). At 5 DPI, skin tissue from the inoculation site was harvested, while at 10 DPI, skin (site of inoculation), heart, and joint tissues were harvested. Genomic DNA was isolated using the DNeasy Blood & Tissue Kit (Qiagen, 69506) according to the manufacturer’s instructions. *B. burgdorferi* burden was quantified by qPCR targeting the *B. burgdorferi flaB* gene, with results normalized to the mouse *β-actin* gene (*Actb*). Results are expressed as *Bb flaB* copies/ 10^5^ mouse *Actb-* copies. Primer sequences are listed in Table 1.

**Table 1:** Primer sequences used in this study.

Gene	Primer Sequence (5’-3’)
*Il6*	F: TACCACTTCACAAGTCGGAGGC R: CTGCAAGTGCATCATCGTTGTTC
*Il1β*	F: TGGACCTTCCAGGATGAGGACA R: GTTCATCTCGGAGCCTGTAGTG
*Tnf*	F: GGTGCCTATGTCTCAGCCTCTT R: GCCATAGAACTGATGAGAGGGAG
*Ifna1*	F: GGATGTGACCTTCCTCAGACTC R: ACCTTCTCCTGCGGGAATCCAA
*Ifnb1*	F: GCCTTTGCCATCCAAGAGATGC R: ACACTGTCTGCTGGTGGAGTTC
*Actin*	F: CATTGCTGACAGGATGCAGAAGG R: TGCTGGAAGGTGGACAGTGAGG
*flaB*	F: CAGCTAATGTTGCAAATCTTTTCTCT R: TTCCTGTTGAACACCCTCTTGA

To assess bacterial viability, tissues were cultivated in BSK-II medium supplemented with phosphomycin (20 μg/mL), rifampin (50 μg/mL), and amphotericin B (2.5 µg/mL) (Sigma-Aldrich). All cultures were incubated at 37 ˚C and monitored for spirochete growth by dark-field microscopy for up to 21 days. A tissue was scored as positive if at least one motile spirochete was detected during the observation period.

### *In vitro* phagocytosis assay

Macrophages (1 × 10^6^ cells/well, 12-well plates) were incubated with GFP-expressing *B. burgdorferi* at an MOI of 10. For all comparisons, the same *B. burgdorferi* preparation was used, and macrophages were exposed to an identical starting inoculum under the same incubation conditions. Where indicated, cells were pretreated for 1 h with recombinant IFN-β (500 pg/mL, Sino Biological Inc., 50708) or an anti-mouse IFNAR1 blocking antibody (1µg/mL, BioLegend, 127322, clone MAR1-5A3), with isotype-matched IgG as a control (BioLegend, 400166, clone MOPC-21). Bacterial uptake was synchronized at 4 °C for 15 minutes, followed by incubation at 37 °C for 2.5 h. In selected experiments, trypan blue (1mg/ml) was added immediately before analysis to quench extracellular GFP fluorescence, thereby distinguishing internalized from extracellular bacteria, as described previously [65]. Mock-treated cells and cells incubated with unlabeled *B. burgdorferi* served as negative controls to set the GFP-negative gate.

To determine whether the effect of IFNAR1 deficiency on bacterial uptake was specific to *B. burgdorferi*, phagocytosis assays were also performed using FITC-*E. coli* according to the manufacturer’s instructions (Cayman Chemical Company, 601370), and bacterial uptake was assessed using the same flow cytometric approach. In all experiments, cells were washed extensively with cold PBS to remove extracellular bacteria, resuspended in FACS buffer (PBS containing 2% FBS), and acquired on a BD Fortessa flow cytometer (BD Biosciences). Macrophages were gated based on forward (FSC) and side scatter (SSC), with cellular debris and doublets excluded. Data were analyzed using FlowJo software (version 10.9.0_CL; BD Life Sciences). Phagocytosis was quantified as the percentage of GFP-positive cells and GFP MFI.

### *In vivo* phagocytosis assay

WT and *Ifnar1^−/^^−^* mice were inoculated intraperitoneally (IP) with GFP-expressing *B. burgdorferi* (1 × 10⁷ spirochetes per mouse) or non-fluorescent *B. burgdorferi* (used to set the GFP-negative gate and control for autofluorescence) for 6 h, as described previously [37]. Peritoneal exudate cells (PECs) were collected and stained with Fixable Viability Dye eFluor 780 (Invitrogen, 65-0865-14), followed by blocking with CD16/32 (BD Pharmingen, 553142) for 15 minutes at 4˚C. The cells were then stained with anti-CD45 (clone 30-F11; BioLegend, 103133), anti-CD11b (clone M1/70; BioLegend, 101215), and anti-F4/80 (clone BM8; BioLegend, 123109) antibodies (Abs) for 30 minutes at 4 ˚C in the dark. After staining, cells were washed and fixed in 1% paraformaldehyde for 15 minutes at RT. Fixed cells were resuspended in FACS buffer and subjected to flow cytometry analysis. Phagocytosis was assessed by determining the percentage of live CD45^+^CD11b^+^F4/80^+^ cells positive for GFP signal. Single-stained, FMO (Fluorescence minus one), and unstained controls were used to set gating parameters and compensation.

### Flow cytometric analysis of phagocytic receptor expression

WT and *Ifnar1^−/^^−^* BMDMs were incubated with *B. burgdorferi* at an MOI of 10 for 2.5 h. Following incubation, cells were washed with cold PBS and stained with antibodies against CD11b (clone M1/70; BioLegend, 101212) and CD14 (clone M14-23; BioLegend, 150105) for 30 min at 4 °C in the dark. Cells were washed, fixed, resuspended in FACS buffer, and acquired on a flow cytometer. Appropriate single-stained, FMO, and unstained controls were used to establish gating parameters and compensation. Surface expression was quantified as the percentage of CD11b⁺ or CD14^+^ cells and the corresponding MFI.

### Confocal microscopy

Macrophages (2 × 10^5^ cells/well, 12-well plates) were seeded on glass coverslips and incubated overnight. The following day, cells were washed with PBS and incubated with GFP-*B. burgdorferi* at an MOI of 10 for 2.5 h. For some experiments, cells were pretreated with recombinant IFN-β (500 pg/mL) or an IFNAR1 blocking antibody (1µg/mL) for 1 h prior to incubation with GFP-*B. burgdorferi*.

To evaluate phagosomal acidification, GFP-*B. burgdorferi* were prelabeled with pHrodo Red succinimidyl ester (Thermo Fisher Scientific, P36600) according to the manufacturer’s instructions prior to incubation with macrophages. Following incubation, cells were washed extensively with ice-cold PBS to remove non-internalized bacteria and fixed with 4% paraformaldehyde for 15 minutes at room temperature. For immunofluorescence staining, fixed cells were blocked in blocking buffer (5% normal goat serum, 0.3% Triton X-100 in PBS) for 1 h. Cells were incubated overnight at 4 °C with primary antibodies targeting Rab5A (Cell Signaling Technology, 2143S) or LAMP1 (Cell Signaling Technology, 99437) to label early and late phagosomal compartments, respectively. After washing, coverslips were incubated with an anti-rabbit Alexa Fluor 594-conjugated secondary antibody (Cell Signaling Technology, 35363) for 2 h at room temperature in the dark. In some experiments, for actin cytoskeletal staining, permeabilized cells were incubated with Phalloidin-California Red Conjugate (Cayman Chemical, 20546) for 1 h at room temperature in the dark. To determine whether the effects of IFNAR1 deficiency on bacterial uptake and phagosomal processing were specific to *B. burgdorferi*, parallel experiments were performed using FITC-*E. coli* (Cayman Chemical Company, 601370).

To further assess intracellular localization of GFP-*B. burgdorferi*, confocal z-stacks were acquired and analyzed using orthogonal views and selected optical z-sections. For quantitative analysis of phagosomal processing, GFP-positive puncta were identified, and the percentages of GFP-positive puncta that were also positive for Rab5A-, LAMP1-, or pHrodo were determined. Coverslips were mounted onto glass slides using VECTASHIELD Antifade Mounting Medium with DAPI (Vector Laboratories, H-1200). Confocal imaging was performed using a Leica upright confocal microscope. Images were acquired using either 20× or 40 × objectives. Images within each experiment were acquired using identical acquisition settings to ensure consistency across samples. Post-acquisition, minor adjustments to brightness and contrast were applied equally to all images within an experiment to enhance visualization without altering the underlying data. Images were analyzed using Fiji and LAS X 3D Viewer.

### Reactive oxygen species (ROS) assay

Intracellular ROS production was assessed using the Highly Sensitive DCFH-DA ROS Assay Kit (Dojindo Laboratories, R252). WT and *Ifnar1^−/^^−^* BMDMs were incubated with *B. burgdorferi* at an MOI of 10 overnight. Next, the cells were washed and incubated with the DCFH-DA working solution for 30 min at 37 °C. Cells were subsequently washed to remove excess probe and acquired on a flow cytometer using the FITC channel. Intracellular ROS production was quantified as the percentage of DCF-positive cells and DCF MFI using FlowJo software.

### Gentamicin protection assay

To assess intracellular killing, a gentamicin protection assay was performed as previously described, with some modifications [66]. Briefly, macrophages were incubated with *B. burgdorferi* for 2.5 h, after which the culture supernatant was collected. Cells were washed gently with ice-cold PBS and resuspended in fresh DMEM containing 10% FBS. Gentamicin (200 µg/mL) was added to the collected supernatant and the macrophages, followed by incubation for 4 h at 37 °C to eliminate extracellular bacteria. To confirm the efficiency of the antibiotic treatment, 100 µL of the treated supernatant was diluted in 20 mL of BSK-II medium and plated in a 96-well plate. To quantify viable intracellular bacteria, the infected macrophages were washed, incubated with sterile pre-warmed distilled water (200 µL) for 5 minutes, and gently lysed via pipetting to release internalized *B. burgdorferi*. A 100 µL of the resulting cell lysate was resuspended in 20 mL of BSK-II medium containing phenol red and kanamycin and plated in 96-well plates. The plates were incubated at 37 ˚C for 21 days. Final growth determination was based on the color change of the medium and confirmed by the presence of motile spirochetes under dark-field microscopy.

### Cytokine analysis by ELISA

To assess cytokine production, BMDMs from WT and *Ifnar1^−/^^−^* mice and hMDMs were incubated with live *B. burgdorferi* at an MOI of 10 for the indicated time points (0, 3, 6, and 24 h). For hMDM experiments, macrophages were pretreated with either an anti-human IFNAR1 antibody (1 µg/mL; InvivoGen, hifnar-mab1) or an isotype control (β-Gal-hIgG1; InvivoGen, bgal-mab1) for 1 h before incubation with *B. burgdorferi*. Following incubation, cell supernatants were collected and centrifuged to remove cellular debris. Concentrations of TNF-α, IL-6, IL-1β, IFN-β, and IFN-α1 in BMDM supernatants, and TNF-α, IL-6, IL-1β, IFN-β and IFN-α2 in hMDM supernatants were quantified using commercial ELISA kits (BioLegend; murine: 430904, 431304, 432604, 447904, 439404; human: 430204, 430504, 437004, 446404, 449504) according to the manufacturer’s instructions.

### Quantitative RT-PCR (qRT-PCR) Analysis

To assess IFN-I expression *in vivo*, WT, *Cgas^−/^^−^*, and *Sting1^−/^^−^* mice were inoculated intradermally (ID) with *B. burgdorferi* (1 × 10⁴ spirochetes per mouse), and *Ifna1* and *Ifnb1* expression was determined in skin tissue from the inoculation site at 5 DPI. To assess inflammatory cytokine expression *in vivo*, WT and *Ifnar1^−/^^−^* mice were similarly inoculated intradermally (ID) with *B. burgdorferi*, and skin tissue from the inoculation site was collected at 5 DPI for cytokine gene-expression analysis. Total RNA was isolated from mouse skin tissues using TRIzol reagent (Thermo Fisher Scientific, 15596018) according to the manufacturer’s protocol. To eliminate genomic DNA (gDNA) contamination, all samples were subjected to on-column digestion using the RNase-free DNase set (Qiagen, 79254), and the RNA was subsequently purified using the RNeasy Mini Kit (Qiagen, 74106). RNA purity and concentration were determined using a NanoDrop One spectrophotometer (Thermo Fisher Scientific). cDNA was synthesized from the RNA template using the PrimeScript 1st strand cDNA Synthesis Kit (Takara Bio, 6110A) according to the manufacturer’s instructions. qPCR was performed in triplicate technical replicates using PowerUp SYBR Green Master Mix (Thermo Fisher Scientific, A25742) on an ABI 7000 Sequence Detection system (Applied Biosystems). The specific primer sequences are detailed in Table 1. The mRNA expression levels of target genes were normalized to the expression of the housekeeping gene *β-actin*. Relative gene expression was calculated using the comparative ΔΔC_T_ method (2^-ΔΔCT^) as described previously [67].

### Immune cell recruitment analysis

To assess immune cell accumulation at the cutaneous inoculation site, WT and *Ifnar1^−/^^−^* mice were inoculated intradermally (ID) with *B. burgdorferi* (1 × 10⁴ spirochetes per mouse). At 5 DPI, mice were euthanized, and skin tissue from the inoculation site was digested with collagenase (1 mg/mL) and DNase (100 µg/mL) for 1 h at 37 °C and processed into single-cell suspensions. Cells were stained with a fixable viability dye, followed by Fc receptor blocking with anti-CD16/32 and staining with anti-CD45 (clone 30-F11; BioLegend, 103133), anti-CD11b (clone M1/70; BioLegend, 101215), anti-F4/80 (clone BM8; BioLegend, 123109), anti-Ly6G (clone 1A8; BioLegend, 127613), and anti-I-A/I-E (clone M5/114.15.2; BioLegend, 107606) antibodies for 30 min at 4 °C in the dark. In some experiments, WT and *Ifnar1^−/^^−^* mice were inoculated intraperitoneally (IP) with *B. burgdorferi* (1 × 10⁴ spirochetes per mouse). At 24 HPI, mice were euthanized, and peritoneal exudate cells (PECs) were stained and processed as described above, except that I-A/I-E staining was not performed. Following staining, cells were fixed and acquired on a flow cytometer. Single-stained, FMO, and unstained controls were used to establish gating parameters and compensation. Data were analyzed using FlowJo software.

### Agonist treatment analysis

For *in vitro* experiments, RAW 264.7 macrophages were pretreated with c-di-AMP (1 µg/mL; InvivoGen, tlrl-nacda) for 1 h before infection with GFP-expressing or non-fluorescent *B. burgdorferi*. The percentage of GFP-positive macrophages and the GFP MFI were determined by flow cytometry. Intracellular bacterial viability was assessed via a gentamicin protection assay as described above.

To elucidate the effect of c-di-AMP *in vivo*, mice were pretreated intradermally (ID) with c-di-AMP (50 µg/mouse; InvivoGen, tlrl-nacda) 24-h before infection with *B. burgdorferi* (1 × 10^4^ spirochetes per mouse). *B. burgdorferi* burden at the site of needle inoculation was determined by qPCR as described above, and the presence of viable bacteria was determined by culturing tissues in BSK-II BAM medium.

### Statistical Analysis

Statistical analyses were performed using Prism (GraphPad Software). All the experiments were performed at least twice. Data are presented as mean ± standard deviation (SD). For comparisons between two groups, statistical significance was evaluated using an unpaired, two-tailed Student’s t-test. For comparisons among three groups, statistical significance was evaluated using one-way ANOVA followed by Sidak’s multiple-comparisons test. For categorical data, statistical comparisons were performed using a two-tailed Fisher’s exact test. *P*-values are represented as *P < 0.05; **P < 0.01; ***P < 0.001; ****P < 0.0001; ns, not significant.

## Supporting information

S1 Fig Validation of intracellular GFP-*B. burgdorferi* localization in BMDMs. **(A)** Representative orthogonal views (XY, XZ, and YZ) of a confocal Z-stack of wild-type (WT) and *Ifnar1^−/^^−^* bone marrow-derived macrophages (BMDMs) incubated with GFP-*B. burgdorferi*. Yellow crosshairs in the XY image indicate the positions used to generate the corresponding XZ and YZ views. Arrows indicate GFP-*B. burgdorferi*. **(B)** Selected optical Z-sections through the same representative WT and *Ifnar1^−/^^−^* BMDMs shown in (A), illustrating the distribution of GFP-*B. burgdorferi* signal through the cellular Z-volume. **(C)** Flow cytometric analysis of GFP-*B. burgdorferi* uptake following trypan blue quenching of extracellular GFP fluorescence. Representative plots and quantification of GFP^+^ BMDMs and GFP mean fluorescence intensity (MFI) are shown. Data are presented as mean ± SD from two independent experiments. Statistical significance was determined using a two-tailed unpaired Student’s *t*-test, ****, *p* < 0.0001.
(TIF)

S2 Fig Type I IFN signaling does not affect macrophage-mediated phagocytosis of *E.coli in vitro.* Bone marrow–derived macrophages (BMDMs) from wild-type (WT) and *Ifnar1^−/^^−^* mice were incubated with FITC-*E. coli* for 2.5 h. (**A**) Flow cytometric analysis of phagocytosis. Representative flow cytometry plots (SSC versus FITC) and quantification of the percentage of FITC-positive cells and FITC mean fluorescence intensity (MFI) are shown. (**B**) Confocal microscopy analysis. Representative confocal images showing FITC-*E. coli* (green) within BMDMs, with quantification of FITC-positive cells. **(C)** Representative confocal images showing FITC-*E. coli* (green) within BMDMs, with quantification of the percentage of LAMP1^+^ puncta among FITC^+^ puncta. Data are presented as mean ± SD from two independent experiments. Statistical significance was determined using a two-tailed unpaired Student’s *t*-test. *, *P* < 0.05; **, *P* < 0.01; ****, *p* < 0.0001.
(TIF)

S3 Fig Type I IFN signaling promotes recruitment of innate immune cells during early *B. burgdorferi* infection. Wild-type (WT) and *Ifnar1^−/^^−^* mice were inoculated intraperitoneally (IP) with *B. burgdorferi* (1 × 10⁴ spirochetes per mouse). Peritoneal exudate cells were harvested at 24 hours post-inoculation (HPI) and analyzed by flow cytometry. (**A**) Flow cytometry gating strategy used to identify macrophages (live CD45^⁺^CD11b^⁺^F4/80^⁺^) and neutrophils (live CD45^⁺^CD11b^⁺^Ly6G^⁺^). (**B**) Quantification of the percentages of macrophages and neutrophils in the peritoneal cavity. Data are presented as mean ± SD (n = 6–8 mice per group) from two independent experiments. Statistical significance was determined using a two-tailed unpaired Student’s *t-*test. *, *P* < 0.05; ***, *P* < 0.001.
(TIF)

## Abbreviations

Bb: borrelia burgdorferi
IFN-I: type I interferon
STING: stimulator of interferon genes
MFI: mean fluorescence intensity
DPI: days post- inoculation
HPI: hours post- inoculation
